# A Tale of 20 Alphaviruses; Inter-species Diversity and Conserved Interactions Between Viral Non-structural Protein 3 and Stress Granule Proteins

**DOI:** 10.3389/fcell.2021.625711

**Published:** 2021-02-11

**Authors:** Gwen Nowee, Julian W. Bakker, Corinne Geertsema, Vera I. D. Ros, Giel P. Göertz, Jelke J. Fros, Gorben P. Pijlman

**Affiliations:** Laboratory of Virology, Wageningen University, Wageningen, Netherlands

**Keywords:** alphavirus, nsP3, hypervariable domain, stress granules, BIN1, G3BP, FMRP

## Abstract

Alphaviruses infect a diverse range of host organisms including mosquitoes, mammals, and birds. The enigmatic alphavirus non-structural protein 3 (nsP3) has an intrinsically disordered, C-terminal hypervariable domain (HVD) that can interact with a variety of host proteins associated with stress granules (SGs). The HVD displays the highest variability across the more than 30 known alphaviruses, yet it also contains several motifs that are conserved amongst different subgroups of alphaviruses. For some alphaviruses, specific nsP3–SG protein interactions are essential for virus replication. However, it remains difficult to attribute general roles to these virus-host interactions, as multiple amino acid motifs in the HDV display a degree of redundancy and previous studies were performed with a limited number of alphaviruses. To better understand nsP3-host protein interactions we conducted comprehensive co-localization experiments with the nsP3s of 20 diverse alphaviruses: chikungunya, Semliki Forest, Sindbis, Bebaru, Barmah Forest, Getah, Mayaro, Middelburg, O'nyong-nyong, Ross River QML and T48, Una, Whataroa, Southern Elephant Seal, Eilat, Tai Forest (TAFV), Venezuelan/Eastern/Western equine encephalitis (V/E/WEEV) and the aquatic Salmonid alphavirus (SAV), with three different SG proteins (G3BP and its insect homolog Rasputin, FMRP) and BIN1 in mammalian and mosquito cell lines. Despite that all terrestrial alphavirus nsP3s contained at least one BIN1-binding motif (PxPxPR), not all nsP3s co-localized with BIN1. Further, all alphaviruses except SAV, TAFV and VEEV displayed co-localization with G3BP. Although viruses lacking FGxF-like motifs contained Agenet-like domain binding motifs to facilitate interaction with FMRP, cytoplasmic nsP3 granules of all tested alphaviruses co-localized with FMRP. Crispr-Cas9 knockout of G3BP in mammalian cells abolished nsP3-FMRP co-localization for all alphaviruses except V/E/WEEV nsP3s that bind FMRP directly. G3BP knockout also changed nsP3 subcellular localization of Bebaru, Barmah Forest, Getah, and Sindbis viruses. Taken together this study paints a more detailed picture of the diverse interactions between alphavirus nsP3 and SG-associated host proteins. The interaction between nsP3 and G3BP clearly plays a central role and results in recruitment of additional host proteins such as FMRP. However, direct binding of FMRP can make the interaction with G3BP redundant which exemplifies the alternate evolutionary paths of alphavirus subgroups.

## Introduction

The *Alphavirus* genus currently contains over 30 members spread all over the world in various hosts and vectors (Strauss and Strauss, 1994; Meshram et al., 2018). Depending on the species, alphaviruses cause rash, arthritis, joint pain, and/or encephalitis in humans and animals resulting in a high disease burden worldwide (Suhrbier et al., 2012; Baxter and Heise, 2020). Although many decades of alphavirus research has generated a comprehensive view on alphavirus structure, replication and host interactions (Fros and Pijlman, 2016; Pietila et al., 2017; Brown et al., 2018; Carpentier and Morrison, 2018; Mendes and Kuhn, 2018), the specific activities of several viral proteins are still not fully understood. Furthermore, the currently available knowledge is primarily based on investigations of only a few key members of the *Alphavirus* genus, i.e., Semliki Forest virus (SFV), Sindbis virus (SINV), chikungunya virus (CHIKV), Ross River virus (RRV), and Venezuelan equine encephalitis virus (VEEV). Some other alphavirus species are emerging, like Mayaro virus (MAYV), Una virus (UNAV), and Getah virus (GETV), whereas others have been known for a long time but were never associated with larger outbreaks or were confined to specific regions, these include Bebaru virus (BEBV), Barmah Forest (BFV), Middelburg (MIDV), O'nyong-nyong (ONNV), Whataroa (WHAV), and Western/Eastern equine encephalitis (W/EEEV).

While the majority of the members in the *Alphavirus* genus is mosquito-borne, there are some notable exceptions. The vector of Southern elephant seal virus (SESV) is most likely a seal louse (La Linn et al., 2001), Salmonid virus (SAV, also known as Salmon pancreas disease virus) has no known vector and can be transmitted horizontally between fish (Moriette et al., 2006), yet SAV can replicate in mosquito cells (Hikke et al., 2014). Finally, Eilat virus (EILV) and Tai Forest virus (TAFV) are insect-specific viruses unable to replicate in vertebrate cells (Nasar et al., 2012; Hermanns et al., 2017). The mosquito-borne alphaviruses have historically been divided into two groups based on the symptoms they cause: arthritogenic and encephalitic alphaviruses (Strauss et al., 1984). However, the genus can also be categorized based on their geographical origin, namely Old World (OW) and New World (NW) alphaviruses in which OW alphaviruses tend to be associated with arthritic symptoms and NW alphaviruses with encephalitic symptoms and more frequently a lethal outcome (Weaver and Frolov, 2005; Suhrbier et al., 2012; Fros and Pijlman, 2016). However, as (alpha)viruses come to be more distributed across the globe, this makes dividing alphaviruses based on its geographic origin no longer preferred. No matter which type of grouping is the most suitable one, several virus-host protein-protein interactions are also specific for parts of the genus.

Alphaviruses are positive-sense, single-stranded RNA viruses with a genome of ~11.5 kb. They mimic cellular mRNAs as they contain both a 5′ cap and a poly(A) tail at the 3′ untranslated region (Strauss et al., 1984). The genome consists of two open-reading frames (ORFs), both encoding polyproteins (Strauss and Strauss, 1994). After non-structural polyproteins are translated, these are cleaved into individual proteins; non-structural protein (nsP) 1, nsP2, nsP3, and nsP4. The functions of nsP1, nsP2, and nsP4 are researched extensively and their role in viral genome amplification during alphavirus replication is therefore established (reviewed in Rupp et al., 2015). Despite a lot of research on nsP3, its exact function still remains relatively unclear (Gotte et al., 2018). NsP3 consist of three domains, the macro domain (Malet et al., 2009), the central zinc-binding alphavirus unique domain (AUD) (Shin et al., 2012), and the hypervariable domain (HVD) (LaStarza et al., 1994; Foy et al., 2013a,b; Rupp et al., 2015). Although both the macro domain and the AUD are highly conserved within the genus, the 150–250 amino acids (aa) long HVD is intrinsically disordered, highly phosphorylated and contains very low levels of amino acid sequence identity between members of the *Alphavirus* genus (Strauss and Strauss, 1994; Rupp et al., 2015). Despite its variability in amino acid sequence and length, the HVD contains several highly conserved domains and aa motifs which are considered to be very specific protein binders.

The HVD in nsP3 is a hub for binding of host proteins that the virus needs for replication or for subversion of host responses (Gotte et al., 2018). Viral infection causes intracellular responses through several related pathways including stress granule (SG) assembly (Lloyd, 2013). SGs are cytoplasmic RNA-protein complexes which assemble as a consequence of translation inhibition. Alphaviruses manipulate the formation of stress granules by interacting with SG components *via* the nsP3 HVD. Two important host proteins known to be sequestered by nsP3 are RasGAP SH3-binding protein (G3BP) and Fragile X mental retardation protein (FMRP). G3BP contains a nuclear transport factor 2 (NTF2)-like domain which is involved in SG formation (Tourriere et al., 2003). Several alphaviruses have mimicked G3BP-binding domains such as FGxF motifs in their HVDs which makes it possible to sequester G3BP and block SG assembly (Cristea et al., 2006; Gorchakov et al., 2008; Fros et al., 2012; Panas et al., 2012, 2014, 2015; Kim et al., 2016). It is hypothesized that some alphavirus nsP3s evolved to have two FGxF motives in order to ensure rapid neutralization of G3BP resulting in SG disassembly (Gotte et al., 2018). The encephalitic alphaviruses (V/E/WEEV) contain an FMRP-interacting domain called the Agenet-like domain binding motif (Kim et al., 2016; Frolov et al., 2017). FMRP is one of three members of the FXR family, which are RNA-binding proteins involved in SG formation (Kim et al., 2016). FMRP redirects bound viral RNA into SGs to suppress translation (Anderson and Kedersha, 2008).

Alphavirus replication initially takes place in invaginations of the plasma membrane, which are normally involved in nascent formation of endosomes and lysosomes (Froshauer et al., 1988; Peranen and Kaariainen, 1991; Spuul et al., 2010). Alphaviruses alter these structures and transform them into spherules with a narrow bottleneck to form replication complexes (Kallio et al., 2013). The protein Bridging Integrator 1 (BIN1, also known as Amphiphysin-II) is one of the key regulators in the early steps of endocytosis, and for some alphaviruses nsP3 was shown to interact with this membrane bending protein *via* proline-rich motifs e.g., PxPxPR (Gotte et al., 2018). BIN1 is essential for efficient viral replication in SFV and SINV (Neuvonen et al., 2011). In addition, the nsP3s of both chikungunya virus (CHIKV) and SFV have a higher affinity for BIN1 than its natural ligand dynamin (Tossavainen et al., 2016). Thus, nsP3 is not only involved in suppressing host stress responses, it also utilizes these host proteins to support its own replication.

Interestingly, alphaviruses that interact with G3BP are considered arthritic alphaviruses and alphaviruses that bind FMRP tend to be encephalitic. The interaction between nsP3 and BIN1 seems to be exclusive for arthritic alphaviruses, but this does not imply that interactions with specific host proteins are directly related to clinical symptoms. The cumulative knowledge on alphavirus nsP3-host protein interactions is mainly based on studies with a select number of alphaviruses. To investigate if these observations also apply to other members in the *Alphavirus* genus, a large subset of 20 alphavirus nsP3s was assessed for their ability to interact with these three host-proteins in four different cell lines and G3BP knockouts thereof through co-localization assays.

## Materials and Methods

### Cell Culture

Vero, HeLa, HeLa G3BP1/2 KO (Visser et al., 2019), U2OS, and U2OS G3BP1/2 KO cells were cultured in Dulbecco's modified Eagle medium (DMEM; Life technologies, Fisher Inv.) containing 10% fetal bovine serum (FBS; Life technologies, Fisher Inv.) and 1% Penicillin/Streptomycin in T25 flasks at 37°C with 5% CO_2_. *Aedes albopictus* mosquito C6/36 cells were cultured in Leibovitz (L15) medium (Gibco) containing 1% non-essential amino acids (NEAAs, Thermo Scientific), 2% tryptose phosphate broth (TPB, Gibco), and 10% FBS. Cells were cultured at 28°C in T25 flasks.

### Plasmid Construction

Alphavirus nsP3 genes were obtained through several methods, including gene synthesis, PCR on cDNA or RT-PCR on purified viral RNA. SuperScript™ III One-Step RT-PCR with Platinum Taq™ (Invitrogen, Fisher Inv.) was performed to amplify the nsP3 gene and to make cDNA from the viral RNA using primers containing AscI (5' end) and NdeI (3' end) restriction sites. PCR products were gel purified using the GE Health care illustra™ GFX^x^ DNA and GEL Band purification kit (GE-Healthcare, Fisher Inv.) and ligated into pGEM®T-Easys-nsP3 (Promega Benelux). The pGEM®T-Easy-nsP3 constructs were electroporated into DH10β competent cells. The nsP3 sequences of EILV, TAFV, and EEEV were synthesized by IDT (gBlocks®) and cloned into pJET1.2/blunt. All nsP3 genes were clones into pDONR207-eGFP downstream eGFP. pDONR207-eGFP-nsP3 plasmids were checked and Gateway cloned into pDEST40 or pUB-GW following the manufacturer's protocol (Life Technologies, ThermoFisher).

### Transfections

All mammalian cell lines were transfected with pDEST40-eGFP-nsP3s. The C6/36 mosquito cells were transfected with both the pPUB-nsP3 constructs and the pPUB-Rin-mCherry. Twenty-four hours prior to transfection, the ~25,000 cells were seeded into eight-well Nunc™ Lab-Tek™ II Chamber Slide™ System (Thermo Fisher Scientific). Transfections of mammalian cells were performed with a total of 400 ng plasmid DNA using the JetPEi®/NaCl system according to manufacturer's protocol (Polyplus-Transfection®). Transfections of mosquito cells were performed with 200 ng plasmid DNA using Fugene HD (Roche).

### Immunofluorescence Assay (IFA)

At 18 h post-transfection (hpt), cells were fixed with 4% paraformaldehyde (PFA; Acros, Fisher Scientific) in PBS for 15 min at room temperature (RT), permeabilized with 0.1% sodium dodecyl sulfate (SDS) in PBS for 10 min at RT and blocked with 5% FBS in PBS for 30 min at RT. Primary antibodies used in this research; mouse-α-BIN1 (1:100; Santa Cruz Biotechnologies); rabbit α-G3BP (1:500; Sigma Aldrich); and rabbit α-FMRP (1:1,000; Abcam), diluted in 5% FBS in PBS and incubated for 1 h at 37°C. The secondary antibodies; goat α-rabbit rhodamine or α-mouse Alexa-Fluor™ 488 (both 1:2,000; Invitrogen), were incubated for 45 min at 37°C and washed three more times. The cells were visualized under standardized conditions, with the same exposure time per experiment (700–1,000 ms) using an Axio Observer Z1m inverted microscope (Zeiss, Jena, Germany) with an X-Cite 120 series lamp. Co-localization percentages were established by counting all transfected cells and determine cytoplasmic granular co-localization. First, cells transfected with the eGFP-nsP3 constructs were identified by green fluorescence. Next, the position of the host protein granules in the transfected cells (G3BP, FMRP, or BIN1, red fluorescence) was compared with that of the nsP3 granules. If several of these granules within the same cell completely overlapped, the cell was scored as positive for co-localization. Cells that were inconclusive for co-localization were scored negative. The percentage co-localization is the number of cells with clear co-localization divided by the total number of transfected cells that were visually analyzed. The images were blinded before they were counted to prevent bias. Between 30 and 120 cells per transfection were counted, depending on the transfection efficiency.

### Phylogenetic Tree

The phylogenetic relationship was determined using the nsP3 coding sequence of the selected viruses (nucleotide sequences in Supplementary Material). SAV was used as an outgroup. Sequences were translated in frame to proteins and aligned using MAFFT version 7 with default settings (Katoh et al., 2019). The protein alignments were converted back into the corresponding codon alignment using PAL2NAL (Suyama et al., 2006). Gblocks (Castresana, 2000) was used for trimming sequences to select conserved domains. PAUP^*^ version 4.0a was used to select the optimal evolution model by critically evaluating the selected parameters. Maximum likelihood analysis (heuristic search, 100 bootstrap replicates) was performed in PAUP, using a submodel of the General Time Reversible Model with invariable sites and a gamma distribution of among-site rate variation (GTR + I + G) with rate class “abcdec.” Bayesian inference was conducted using MrBayes 3 (Ronquist and Huelsenbeck, 2003), using the GTR + I + G model. Analyses were initiated from random starting trees. Two separate Markov chain Monte Carlo (MCMC) runs, each composed of four chains (one cold and three heated), were run for 6,000,000 generations. The cold chain was sampled every 100 generations, and the first 15,000 generations were later discarded (a burn-in of 25%). Posterior probabilities were computed from the remaining trees.

## Results

### Identification of Stress Granule Protein (G3BP and FMRP) and BIN1-Binding Motifs in the Alphavirus nsP3 Hypervariable Domain

**Figure 1:**
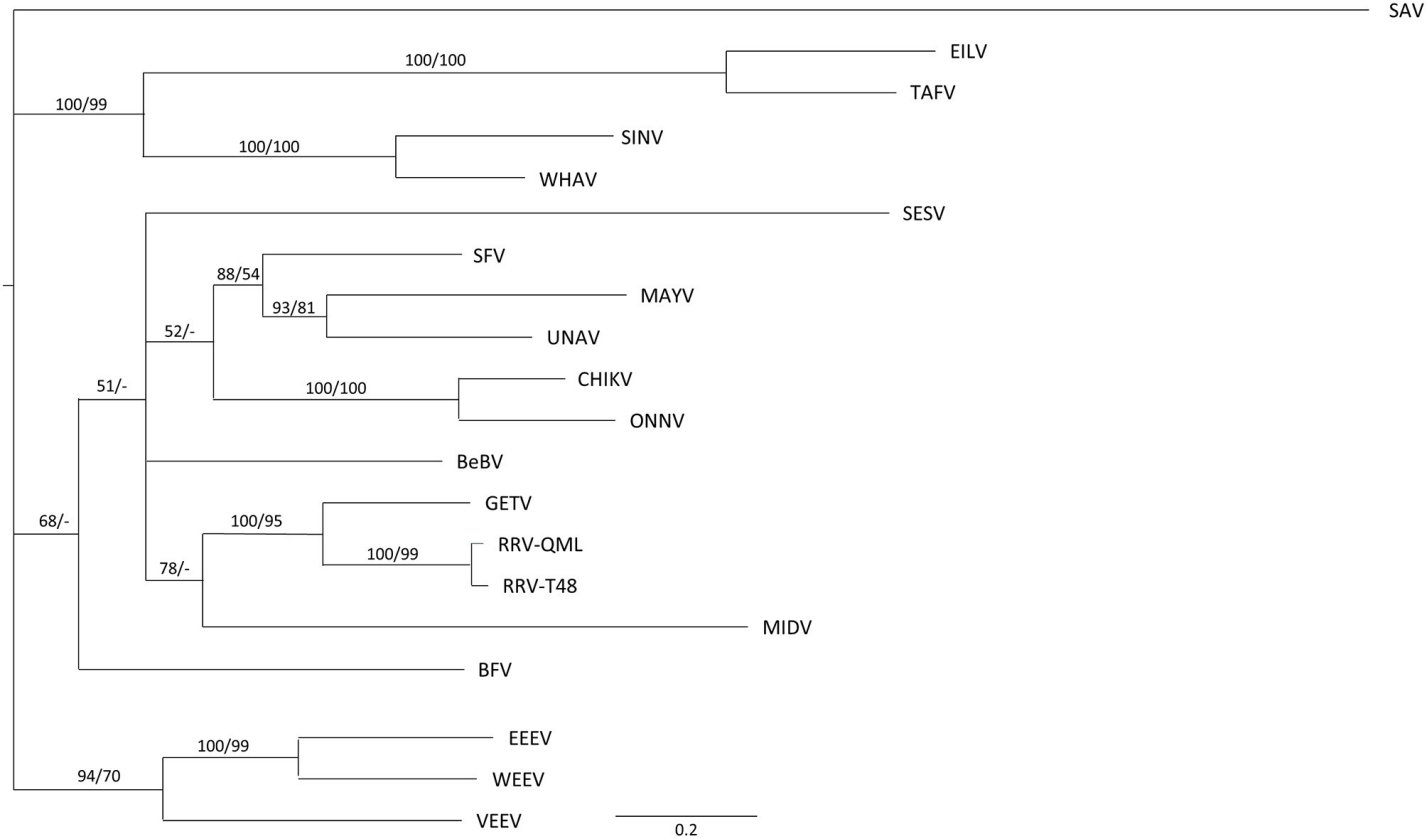
Evolutionary relationships between alphavirus nsP3 sequences. A Bayesian phylogeny of alphavirus nsp3 coding sequences is shown a GTR + I + G model. Bayesian posterior probabilities and maximum likelihood values (100 replicates) are shown before and after the slash respectively. The scale bar represent 0.02 expected substitutions per site. A dash (-) indicates values <50.

The first step in the analysis of alphavirus nsP3 interactions with host proteins was to identify the putative G3BP, FMRP, and BIN1-binding motifs of the 20 alphaviruses in our study. A phylogenetic tree based on nsP3 amino acid sequences shows several clades within the *Alphavirus* genus (Figure 1). As most alphavirus phylogenetic trees in literature are based on complete genomes, the structural proteins or the RdRp, this phylogenetic tree reveals some unexpected relations. For example, nsP3 of SINV and WHAV cluster with that of the insect-specific alphaviruses TAFV and EILV.

**Figure 2:**
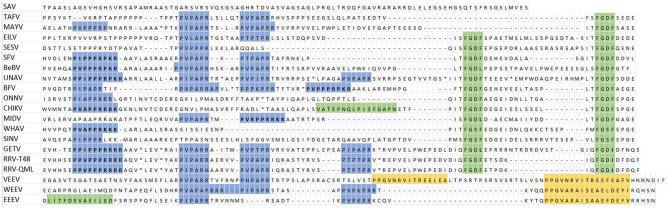
Part of an alignment of 20 alphavirus nsP3 HVDs based on three host protein-binding motifs. In blue; the proline-rich BIN1-binding motif, PxPxPR. In blue and bold; high affinity proline-rich motifs, P[I/V][P/A]PPR[R/K/P][R/K][R/K]. In yellow; Agenet-like domein binding motif which interacts with FMRP. In green; G3BP-binding motifs, the G3BP non-conicial motif (CHIKV), the LITF motif (EEEV), and the FGDF motifs. In green and bold, the fourth residue of the FGDF motifs is either an isoleucine or leucine. (*) part of the alignment not shown.

Next, an amino acid alignment of the nsP3 HVD was made guided by the binding motifs of G3BP, FMRP, and BIN1 (Figure 2). All alphaviruses, except SAV and V/W/EEEV, contain at least one G3BP-binding motif (Figure 2, green highlight). Most alphaviruses contain the canonical FGxF motif, but in some motifs the second phenylalanine is substituted by isoleucine or leucine, *e.g*., BEBV (FGDL), GETV (FGDI and FGDL), and SINV (FGSF and FGDI). Therefore, all FG[D/S][I/L/F] motifs will be referred to as FGDF-like motif. Additional non-canonical G3BP-binding motifs are also shown in the alignment. As expected, the Agenet-like domain binding motif, which is involved in binding of FMRP, was only found in the V/W/EEV clade (Figure 2, yellow highlight), with two of these domains present in VEEV. The PxPxPR motif (Figure 2, blue highlight) is a conserved motif within the HVD of nsP3 and constitutes the BIN1-binding site of nsP3, although additional proline-rich motifs may also interact with BIN1. The proline-rich motif with the highest binding affinity to BIN1 was suggested to be P[I/V][P/A]PPR[R/K/P][R/K][R/K] (Tossavainen et al., 2016), and can be found in the nsP3s of BEBV, CHIKV, GETV, SFV, UNAV, and both strains of RRV. In an IFA experiment, we observed co-localization of many alphavirus nsP3s with BIN1 in cytoplasmic granules to a certain degree (Supplementary Figures 1, 2). SAV represents an alphavirus outgroup (Figure 1) and lacks all the binding motifs studied in this research (Figure 2).

### Most Alphavirus nsP3s Co-localize With G3BP and Mosquito Rasputin in Cytoplasmic Granules

**Figure 3:**
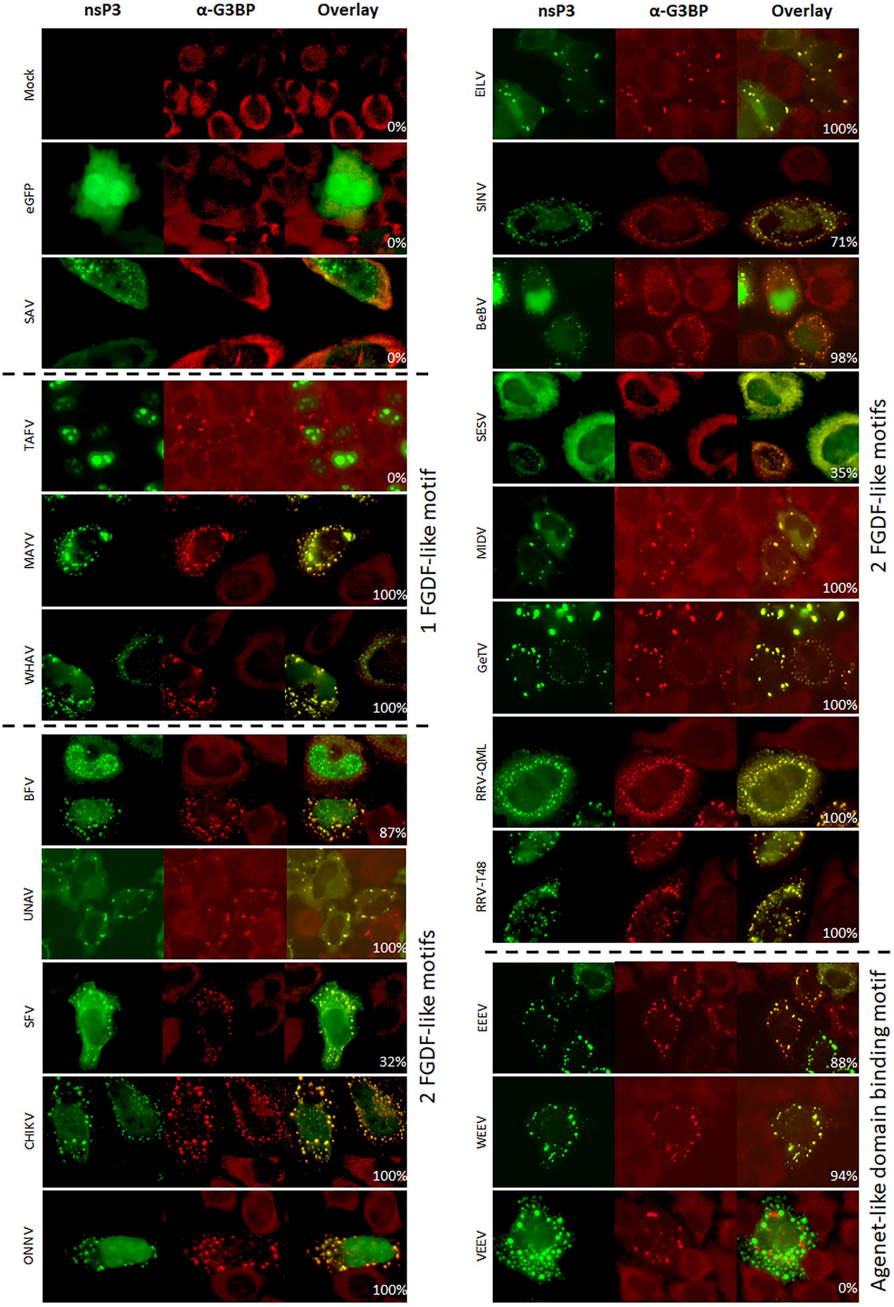
Alphavirus nsP3 co-localization with G3BP. HeLa cells were transfected with alphavirus nsP3-eGFP, stained with antibodies for G3BP and visualized by immunofluorescence. Green indicates localization of nsP3, red represents G3BP. NsP3-G3BP co-localization is visualized in yellow in the overlay. The percentages in the overlay images represent co-localization percentage of all counted cells. A distinction is made for alphaviruses with one or two FGDF-like motifs or the presence of one or two Agenet-like domain binding motifs.

**Figure 4:**
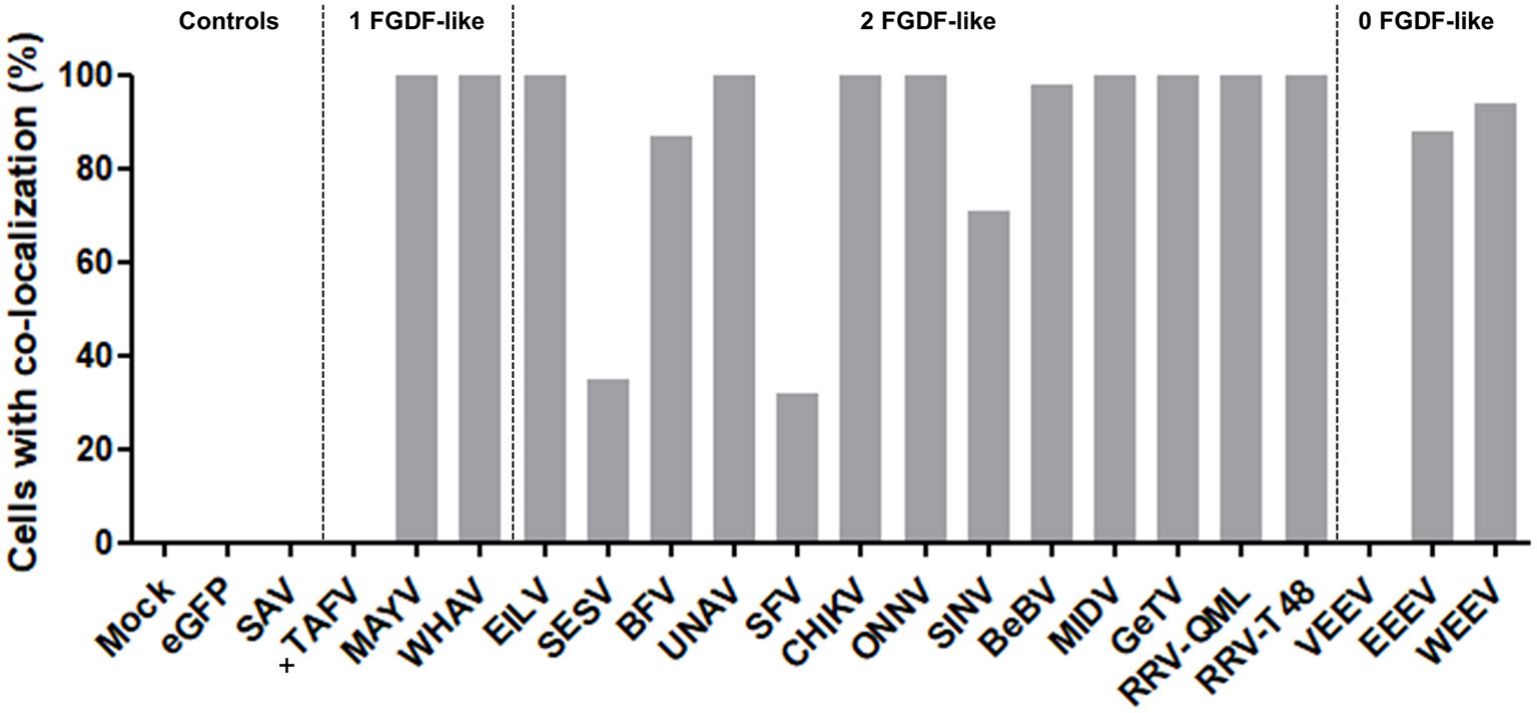
NsP3-G3BP co-localization percentages. HeLa cells with co-localization of nsP3 and G3BP are represented as co-localization percentages. Bars represent the mean co-localization pattern of 30–50 transfected HeLa cells. A distinction is made for alphaviruses with zero, one, or two FGDF-like motifs. In absence of cytoplasmic granules, no co-localization could be determined, these nsP3-eGFPs are indicated with (+).

The co-localization of alphavirus nsP3s with G3BP was investigated using IFA in HeLa cells (Figure 3) and in U2OS cells (Supplementary Figure 3). Clear nsP3 granules were observed for almost all alphavirus nsP3s, except for TAFV-nsP3 which displayed exclusive localization to the nucleus (Figure 3). MIDV-nsP3 and UNAV-nsP3 showed clear cytoplasmic granules in HeLa cells, but had a diffuse localization in U2OS cells (Supplementary Figure 3). NsP3-G3BP granular co-localization was quantified per transfected cell and represented as percentages (Figure 4, Supplementary Figure 4). All 20 alphavirus nsP3s were grouped based on the number of FGDF-like motifs. Interestingly, almost every alphavirus-nsP3 co-localized with G3BP except SAV and VEEV. The nsP3s of EILV, BFV, CHIKV, ONNV, WHAV, BEBV, MAYV, GETV, RRV^QML^, RRV^T48^, EEEV, and WEEV all showed nearly 100% co-localization with G3BP. No major differences between HeLa and U2OS cell line with regard to nsP3-G3BP co-localization percentages was observed (Supplementary Figure 4). Nevertheless, SFV-nsP3 showed some variety between the cell lines, with a 30% G3BP co-localization in HeLa cells and a 85% in U2OS cells. Interestingly, WEEV-nsP3 does not contain a known G3BP-binding motif (Figure 1) but did co-localize with G3BP.

**Figure 5:**
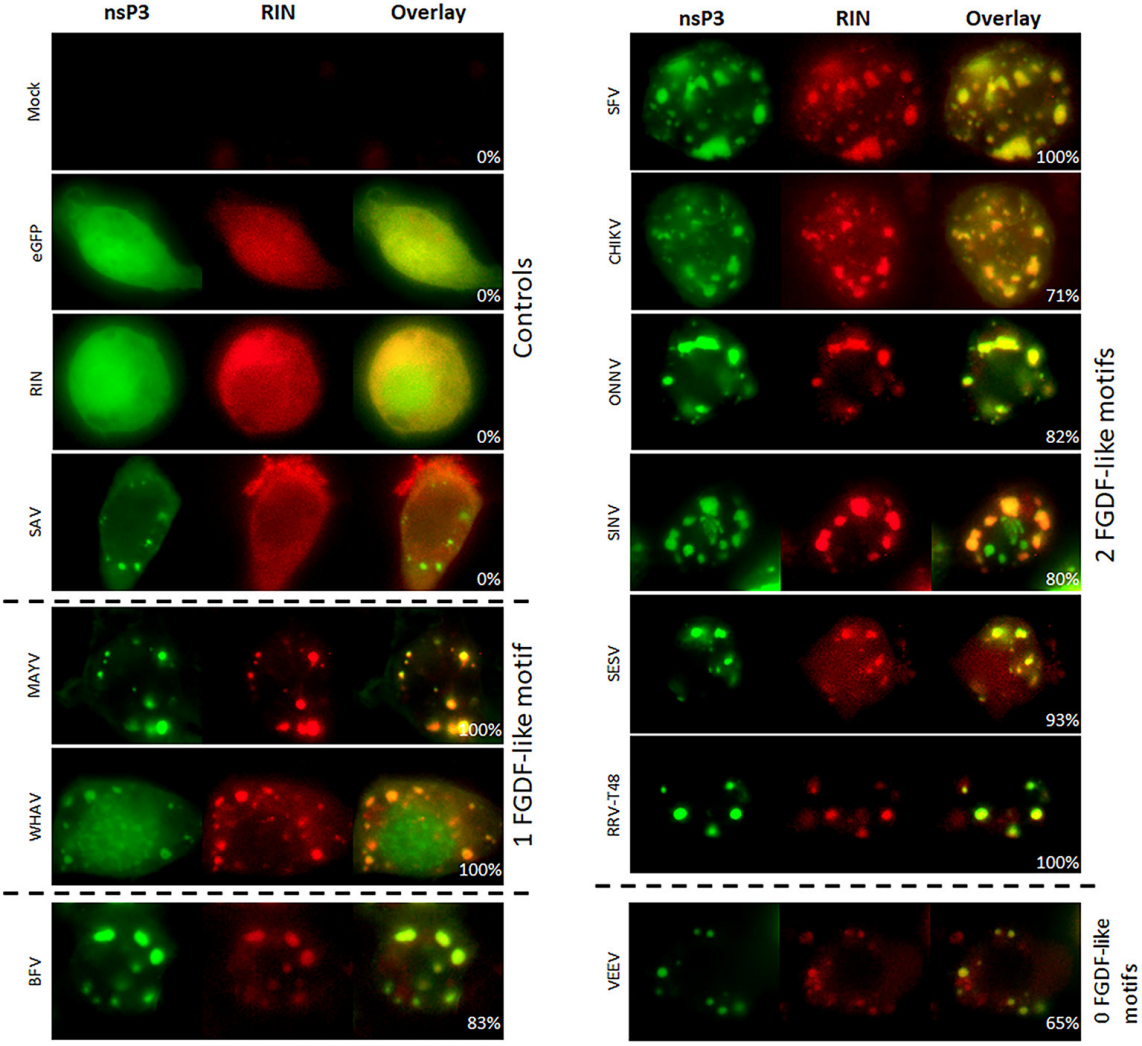
Alphavirus nsP3 co-localization with RIN in mosquito cells. C6/36 cells were transfected with alphavirus nsP3-eGFP and pPUB-RIN. Green indicates localization of nsP3, red represents RIN. NsP3-RIN co-localization is visualized in yellow in the overlay. The percentages in the overlay images represent co-localization percentage of all counted cells. A distinction is made for alphaviruses with zero, one, or two FGDF-like motifs.

**Figure 6:**
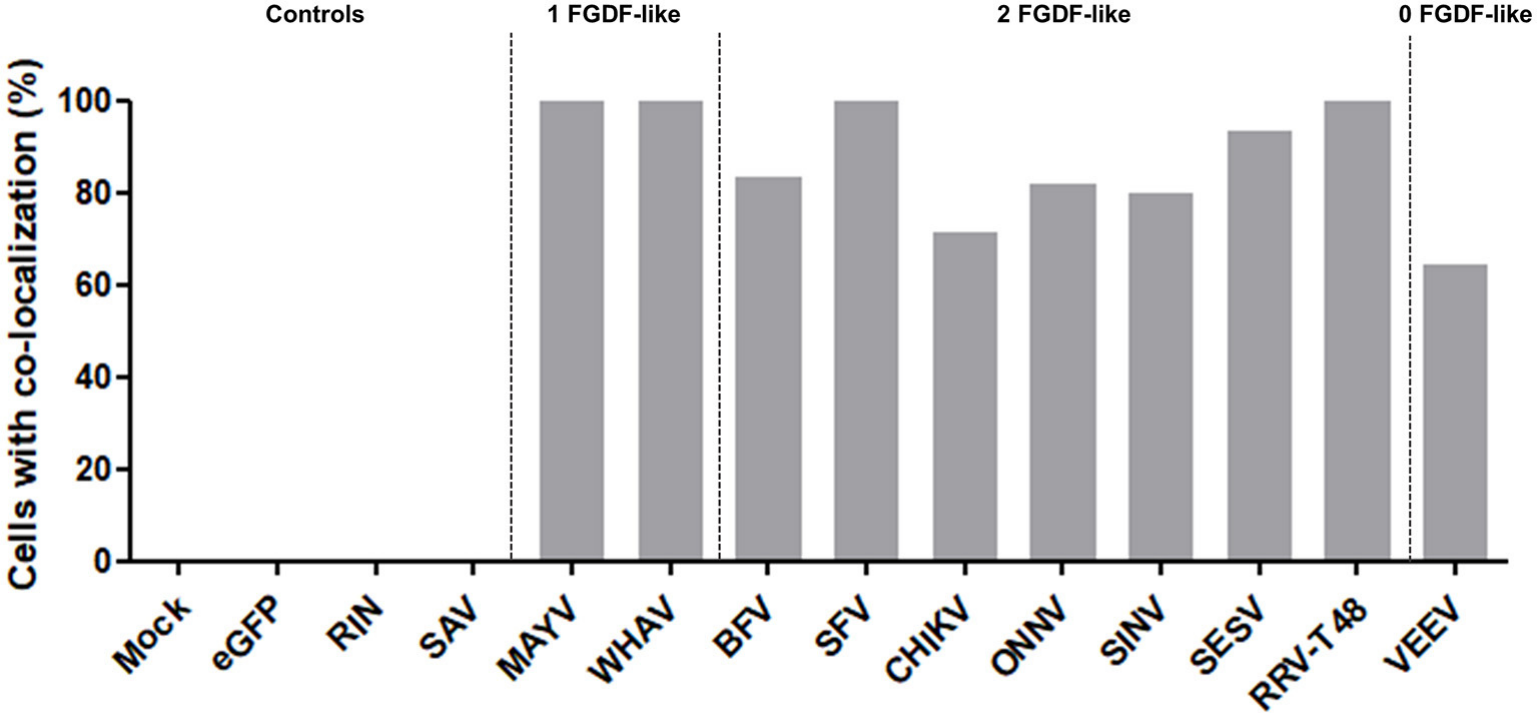
NsP3-RIN co-localization percentages in mosquito cells. Cells with co-localization of nsP3 and RIN are represented as co-localization percentages. Bars represent the mean co-localization pattern of 50–100 transfected C6/36 cells. A distinction is made for alphaviruses with zero, one, or two FGDF-like motifs.

The interaction between nsP3 and G3BP was also investigated in mosquito cells. The mosquito homolog of G3BP is Rasputin (Rin), but due to lack of specific antibodies, Rin was expressed as fusion protein to mCherry (Fros et al., 2015a). The co-localization studies were conducted with a subset of nsP3 constructs representing the different clades. All tested nsP3s displayed granular localization in mosquito cells. In contrast, singly expressed Rin-mCherry displayed a diffuse localization throughout the cell (Figure 5). When nsP3 and Rin were simultaneously expressed, both RIN and nsP3 co-localized in cytoplasmic granules for all alphavirus nsP3s except that of SAV. The level of co-localization does not directly correlate with the number of FGDF domains, since MAYV and WHAV nsP3s displayed a very high level of co-localization with Rin despite only carrying a single FGDF-like domain (Figure 6). Surprisingly, VEEV nsP3 (no predicted G3BP-binding motif) also co-localized with Rin in about 60% of cells, although co-localization within individual cells was not always complete (Figure 5).

### Alphavirus nsP3-FMRP Co-localization Can Be Mediated by G3BP

**Figure 7:**
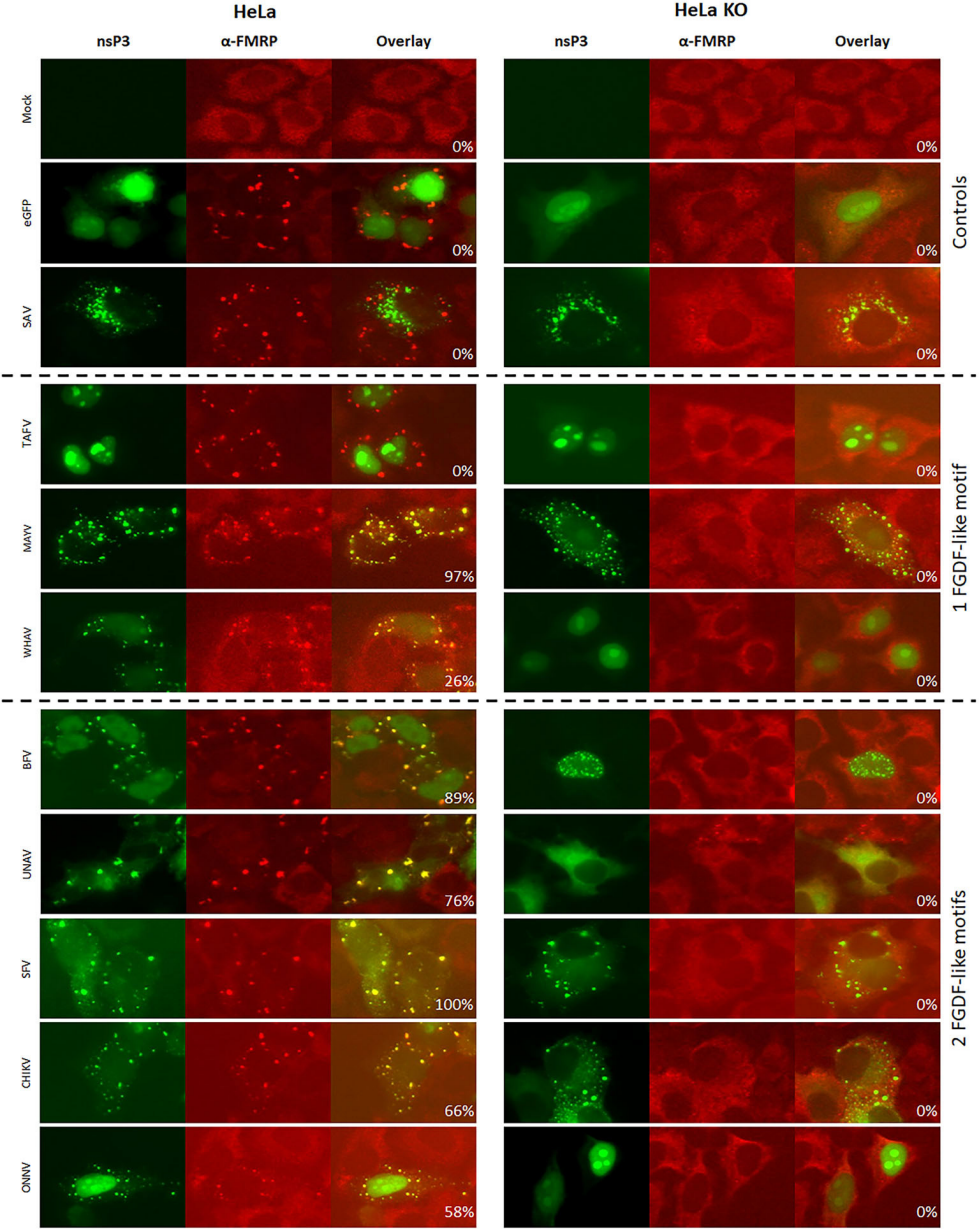
Alphavirus nsP3 co-localization with FMRP in wildtype and G3BP KO cells. HeLa wt and HeLa G3BP KO cells were transfected with alphavirus nsP3-eGFP, stained with antibodies for FMRP and visualized by immunofluorescence. Green indicates localization of nsP3, red represents FMRP. NsP3-FMRP co-localization is visualized in yellow in the overlay. The percentages in the overlay images represent co-localization percentage of all counted cells. A distinction is made for alphaviruses with one or two FGDF-like motifs or the presence of one or two Agenet-like domain binding motifs.

**Figure 8:**
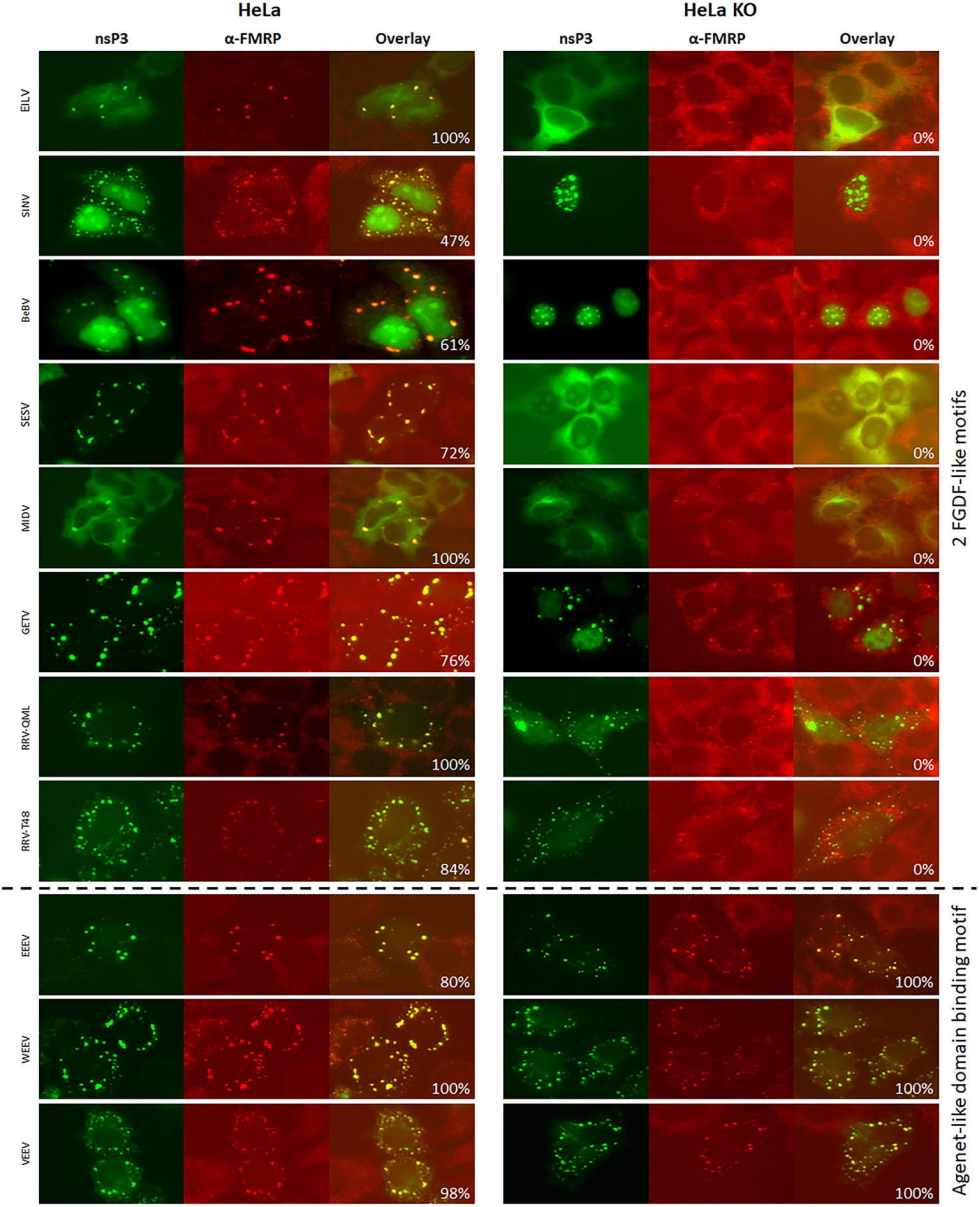
Alphavirus nsP3 co-localization with FMRP in wildtype and G3BP KO cells. HeLa wt and HeLa G3BP KO cells were transfected with alphavirus nsP3-eGFP, stained with antibodies for FMRP and visualized by immunofluorescence. Green indicates localization of nsP3, red represents FMRP. NsP3-FMRP co-localization is visualized in yellow in the overlay. The percentages in the overlay images represent co-localization percentage of all counted cells. A distinction is made for alphaviruses with one or two FGDF-like motifs or the presence of one or two Agenet-like domain binding motifs.

To further investigate the differences in the interaction with SG proteins between the 20 alphaviruses, the co-localization of nsP3 with FMRP was examined. On the basis of existing knowledge, it was hypothesized that nsP3s with an Agenet-like domain binding motif would display strong co-localization with FMRP (Frolov et al., 2017). Indeed, very high co-localization percentages (80–100%) were observed between V/W/EEEV-nsP3 and FMRP in HeLa cells (Figures 7, 8). In U2OS cells, the co-localization between W/EEEV-nsP3 and FMRP was again high, but not for VEEV-nsP3 (Supplementary Figures 5, 6).

**Figure 9:**
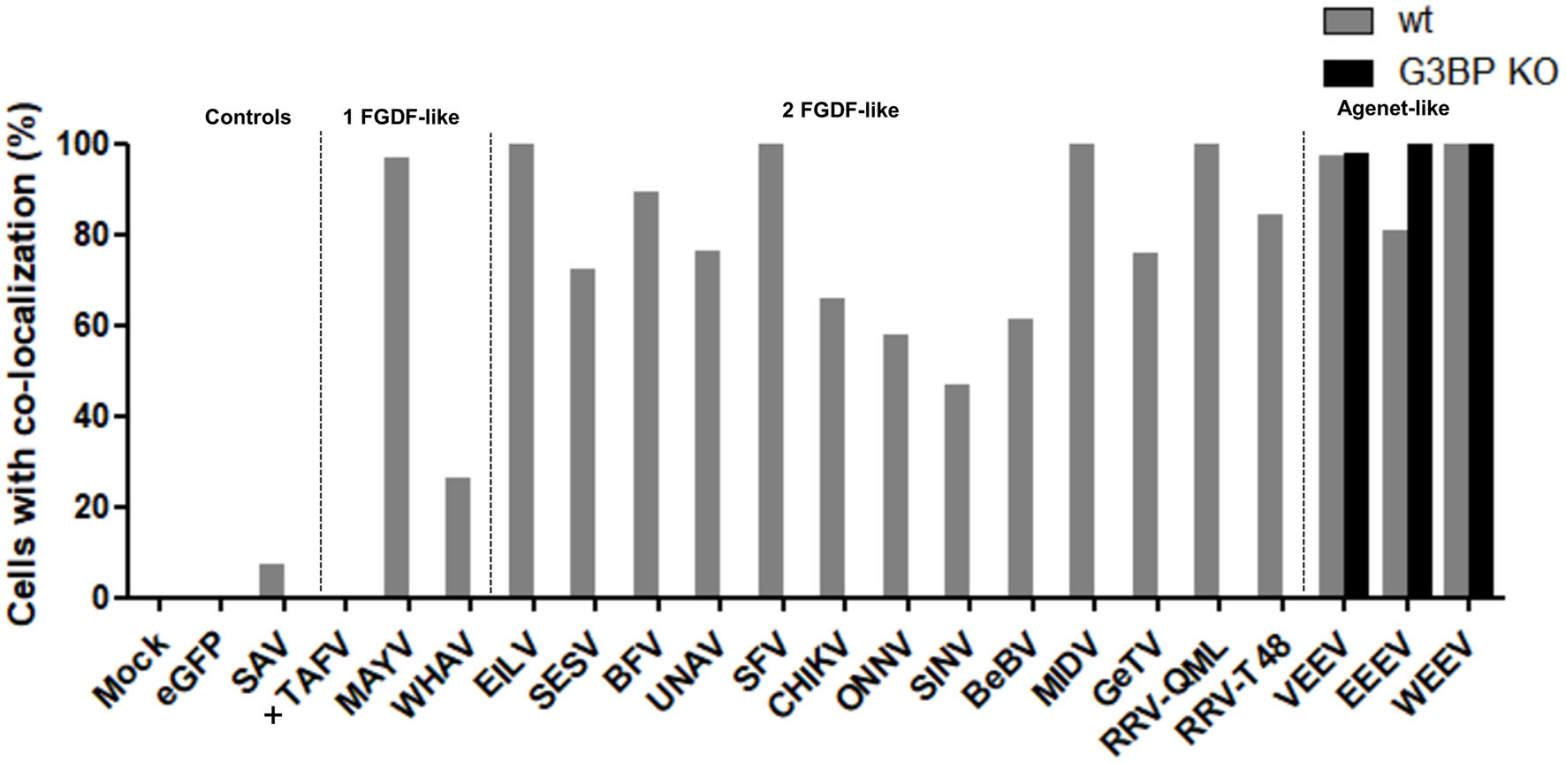
NsP3-FMRP co-localization percentages in wildtype and G3BP KO cells. Cells with co-localization of nsP3 and FMRP are represented as co-localization percentages. Bars represent the mean co-localization pattern of 50-100 transfected HeLa wt and HeLa G3BP KO cells. A distinction is made for alphaviruses with one or two FGDF-like motifs or the presence of one or two Agenet-like domain binding motifs. In absence of cytoplasmic granules, no co-localization could be determined, these nsP3-eGFPs are indicated with (+).

Interestingly, all other observed alphavirus nsP3 granules also contained FMRP (Figures 7, 8), despite the lack of an Agenet-like domain binding motif in these nsP3s (Figure 2). One of the possibilities is that FMRP co-localization with these alphavirus nsP3s is mediated by G3BP. To experimentally address this possibility, the co-localization between nsP3 and FMRP was compared between wildtype and G3BP knock-out (KO) cell lines (Figure 7). As expected, V/W/EEEV-nsP3s were still able to interact with FMRP and co-localization was retained (Figure 8), suggesting that this interaction was independent of G3BP. For the other alphavirus nsP3s, which lack the Agenet-like domain binding motif (BFV, BEBV, CHIKV, EILV, GETV, MAYV, MIDV, ONNV, RRV^QML^, RRV^T48^, SESV, SFV, SINV, UNAV, and WHAV), it was observed that the absence of G3BP completely abolished co-localization with FMRP (Figure 9). The results obtained in HeLa G3BP KO cells were very similar to those obtained in U2OS G3BP KO cells (Supplementary Figures 5, 6). We conclude that alphavirus nsP3s without an Agenet-like domain binding motif can co-localize with FMRP in a G3BP-dependent manner.

### NsP3 Localization Alters in the Absence of G3BP

During the study on the interactions of the nsP3 proteins of 20 different alphaviruses with specific SG proteins in host cells, several additional observations were made. First, the nsP3 localization phenotype of some alphaviruses appeared to be dependent on the cell type. For example, several nsP3s localized in the nucleus whilst others localized in the cytoplasm in both a granular and a diffuse manner. Furthermore, for multiple alphavirus nsP3s, their localization altered in absence of G3BP (Figure 9 and Supplementary Figure 6). Clearly, the cytoplasmic granular localization of nsP3s of BEBV, GETV, BFV, SINV, WHAV, and SESV was dependent on the presence of G3BP. Further, the nsP3s of BEBV, BFV, SINV, and WHAV nsP3s localized exclusively to the nucleus when cells lacked G3BP, whereas in wild-type cells the nsP3 signal was mainly cytoplasmic granular and/or nuclear diffuse (Figures 3, 5, 7, 8 and Supplementary Figure 5). Finally, SESV-nsP3 had a cytoplasmic granular localization in wild-type cells, but in the absence of G3BP, the protein was observed diffusely in the cytoplasm and in the nucleus.

## Discussion

In the present study the interaction between alphavirus nsP3s and SG proteins G3BP and FMRP and host protein BIN1 was investigated. We observed that V/W/EEEV-nsP3 did not readily co-localize with BIN1 despite having a putative binding motif, while all alphaviruses with PxPxPR and FGDF-like motifs were able to co-localize with BIN1. We found that alphavirus nsP3s containing an FGDF-like motif co-localized with G3BP and that the nsP3s containing the Agenet-like domain binding motif co-localized with FMRP. Furthermore, the granular localization of several nsP3s was shown to depend on G3BP.

The interactions between the nsP3 HVD and SG proteins play important roles during alphavirus replication. Cytoplasmic granules that optically resembled SGs (but were not *bona fide* SG as they lacked other canonical SG markers such as eIF3) lost their functionality in the presence of CHIKV and SFV nsP3 (Fros et al., 2012; Panas et al., 2012). The nsP3-G3BP interaction was not only an effective means to limit SG-mediated antiviral responses, the interaction of nsP3 with G3BP proved crucial for establishing virus replication (Scholte et al., 2015). Similarly, the mosquito protein and G3BP homolog Rasputin was readily sequestered to nsP3 granules in mosquito cells (Figure 5). We previously showed that the FGDF motifs of CHIKV-nsP3 and their interaction with mosquito Rasputin are crucial for effective infection and dissemination of CHIKV in mosquito vectors (Fros et al., 2015a; Goertz et al., 2018a). In vertebrate cells, the interactions between the nsP3 molecules of SINV, CHIKV, and SFV with SG proteins G3BP1/2 have been studied in most detail (reviewed by Gotte et al., 2018). It was hypothesized that all alphaviruses nsP3s containing at least one FGDF-like motif in their HVD would co-localize with G3BP. Indeed, the nsP3s of BFV, BEBV, CHIKV, EILV, GETV, MAYV, MIDV, ONNV, RRV^QML^, RRV^T48^, SESV, SINV, UNAV, and WHAV showed co-localization with G3BP in transfected cells (Figure 3). The exception was TAFV-nsP3, which has 1 FGDF motif but localized exclusively to the nucleus. In the TAFV-nsP3 expressing cells, G3BP, and FMRP are present in discrete cytoplasmic granules, suggesting that *bona fide* SGs are induced, perhaps in response to overexpression or protein misfolding. It should be noted that TAFV is an insect-specific virus, which means that the behavior of TAFV proteins in mammalian cells may be less relevant.

Not all alphavirus HVDs have exact FGDF motifs. For example, GETV-nsP3 contains two FGDF-like motifs of which the fourth residue is either an isoleucine or a leucine. The second phenylalanine of the FGDF motif of SFV was identified as being essential for binding with G3BP, however this conclusion was based on an amino acid change of phenylalanine to alanine (Panas et al., 2015). The results obtained in this study indicate that GETV-nsP3 granules sequester G3BP even though GETV HVD contains FGDI and FGDL domains. V/W/EEV do not contain an FGDF-like motif, however EEEV-nsP3 contains an LITF motif which can bind G3BP (Frolov et al., 2017), and indeed co-localized with G3BP in our study. WEEV-nsP3 lacks both the FGDF-like and LITF motifs but did show co-localization with G3BP, whereas VEEV-nsP3 and G3BP did not co-localize. Whether WEEV-nsP3 directly interacts with G3BP *via* an unknown binding motif (or perhaps more likely through indirect interactions) remains to be determined.

Instead of containing dedicated FGDF-like domains to bind G3BP, V/W/EEV-nsP3 have one or more Agenet-like domain binding motifs. The V/W/EEV nsP3s granules indeed showed almost 100% co-localization with cellular FMRP. Granular localization of all other alphavirus nsP3s resulted in significant co-localization with FMRP. However, this co-localization was completely abolished when G3BP was lacking, indicating that sequestration of FMRP to these nsP3 granules was mediated by an indirect interaction that required G3BP (Figure 9). Together this suggests interactions between nsP3 and G3BP or FMRP protein families contain a degree of redundancy. Indeed, the only virus that contains confirmed binding domains for both G3BP and FMRP, EEEV, redundantly uses these cellular proteins to effectively replicate its RNA (Frolov et al., 2017).

Many of the alphavirus nsP3s multimerize into cytoplasmic granules both in the presence and absence of G3BP (Figure 3). Some nsP3 molecules however displayed a more variable subcellular localization, i.e. BEBV-, BFV-, ONNV-, SINV-,TAFV-, and WHAV-nsP3 were also observed in the nucleus of transfected cells (Figure 3). The HVD of BEBV-nsP3, BFV-nsP3, and WHAV-nsP3 contains a predicted nuclear localization signal (NLS) which likely causes the nuclear localization (Lange et al., 2007; Kosugi et al., 2009a). In addition to importin α-mediated nuclear import that requires the imported protein to contain a classical NLS, other means of nuclear import exist that may be utilized by other alphavirus nsP3s. The NTF2 domain of G3BP is involved in nuclear transport and binds with nucleoporin p62 (Nups62) *via* an FxFGxF motif to facilitate nuclear import (Clarkson et al., 1996; Kosugi et al., 2009b). This motif is mimicked by the nsP3s of TAFV, BFV, and WHAV and can interact with several importins. Additionally, an FG repeat motif can be recognized by the TAP/p15 heterodimer which may also result in nuclear import (Fribourg et al., 2001). Repeats of three FG motifs are found in the HVD of BEBV, MAYV, BFV, CHIKV, MIDV, and SINV. It is not easy to attribute a biological role for nsP3 nuclear localization, but other alphavirus gene products, i.e., capsid and nsP2, also translocate to the nucleus to block the nuclear pore complex, shut down transcription or block JAK-STAT signaling by promoting STAT1 nuclear export (Atasheva et al., 2008; Fros et al., 2013; Goertz et al., 2018b). Defining the role of nsP3 in these, or other, processes requires more experimentation.

Interestingly, in the absence of G3BP, several alphavirus nsP3s showed a different subcellular localization (Figure 7). G3BP is essential for SG assembly triggered by eukaryotic initiation factor 2α (eIF2α) phosphorylation, which means that SGs can no longer form in the absence of G3BP (Gotte et al., 2018). CHIKV nsP3 granules are negative by IFA for the SG marker eIF3, suggesting that nsP3 granules are not *bona fide* SGs, but rather aggregates of nsP3 that effectively sequester G3BP (Fros et al., 2012). Whether eIF2α is phosphorylated in cells expressing nsP3 cytoplasmic granules has not been investigated, but seems unlikely as this requires activation by PKR, or by PERK in the unfolded protein response (Fros et al., 2015b; Fros and Pijlman, 2016). The partly cytoplasmic signal of BEBV-, SINV-, and SFV-nsP3 was completely abolished and only nuclear nsP3 was observed when expressed in G3BP knockout cells. This suggests that G3BP and host proteins involved in nuclear transport compete for the alphavirus FxFGxF or FG-repeats in the HVD of nsP3. But again, different phenotypes are observed for other alphaviruses, i.e., the nsP3s of CHIKV, MIDV, and MAYV were not observed to translocate into the nucleus despite having a FG-repeat. Thus, whether or not nuclear localization of nsP3 plays is important for alphavirus infection remains an intruiging topic for further investigation.

Taken together, this study expands the current knowledge on alphavirus nsP3-host protein interactions. Most notably, all mosquito-borne alphaviruses interact with SG components. Even though WEEV, VEEV, and EEV clearly followed a different evolutionary path, their interaction with FMRP and other FXR family proteins seems functionally homologous to the interaction between G3BP1/2 and the nsP3s of other members of the genus.

## Data Availability Statement

The datasets presented in this study can be found in online repositories. The names of the repository/repositories and accession number(s) can be found in the article/Supplementary Material.

## Author Contributions

GG, JF, and GP designed the study. GN, JB, and CG executed the experiments. GN and JB prepared the figures. VR prepared the phylogenetic tree. GN wrote the first draft. JF and GP wrote the final version of the manuscript. All authors contributed to the article and approved the submitted version.

## Conflict of Interest

The authors declare that the research was conducted in the absence of any commercial or financial relationships that could be construed as a potential conflict of interest.
