# Supplementary material for: A Tale of 20 Alphaviruses; Inter-species Diversity and Conserved Interactions Between Viral Non-structural Protein 3 and Stress Granule Proteins
Source: Front Cell Dev Biol. 2021 Feb 11;9:625711. doi: 10.3389/fcell.2021.625711 (PMC7905232; doi:10.3389/fcell.2021.625711)
Supplement: Supplementary file 1 [file Data_Sheet_1.pdf]

frontiers

Supplementary Material

Alphavirus nsP3 co-localization with BIN1 in cytoplasmic granules

While all alphavirus nsP3s (except SAV) have at least one proline-rich motif (**Figure 2**), it was experimentally confirmed that CHIKV and SINV can bind BIN1 whereas EEEV and VEEV cannot (Frolov et al., 2017). To investigate co-localization of nsP3 of additional alphaviruses with BIN1, the nsP3 proteins of twenty alphaviruses fused to eGFP were transiently expressed in mammalian cells. The cells were fixed and stained for BIN1 by IFA (**Figure S1**). NsP3-BIN1 granular co-localization was quantified and represented as percentage cells with observed co-localization. All twenty alphavirus nsP3s were grouped based on the number of proline-rich motifs and high-affinity proline-rich motifs are indicated with an asterisk (**Figure S2**).

Many alphavirus nsP3s localized with BIN1 in cytoplasmic granules to a certain degree. A high percentage of nsP3-BIN1 co-localization (>50%) was observed for BFV, BEBV, MAYV, GETV and RRV^{QML} (**Figure S2**). Moderate nsP3-BIN1 co-localization (20-50%) was observed for SESV-, CHIKV-, ONNV-, SINV-, and RRV^{T48}. The nsP3s of SAV, VEEV, EILV, EEEV, SFV and WHAV showed very low to no co-localization with BIN1. TAFV nsP3 showed nuclear localization and MIDV and UNAV nsP3 showed diffuse cytoplasmic localization, therefore nsP3-BIN1 co-localization in cytoplasmic granules could not be determined for these viruses (**Figure S1**). A clear division was observed regarding the ability of nsP3s to co-localize with BIN1. All alphavirus nsP3s that also contained an FGDF-like motif co-localized with BIN1 to a certain extent. In contrast, alphavirus nsP3s with an Agenet-like domain binding motif did not co-localize with BIN1 at all, despite the presence of a proline-rich motif.

The HVD of nsP3 contains several highly conserved motifs of which PxPxPR is often present multiple times. A previous study reported that all alphavirus nsP3s contain this motif accept those of African origin (Aaskov et al., 2011). However, all alphaviruses in our study including those of African origin (except SAV) contain at least one copy of the PxPxPR motif (**Figure 2**). Co-immunoprecipitation of BIN1 with SINV and CHIKV nsP3 indicated direct binding between these molecules (Frolov et al., 2017). Here we showed that most alphavirus nsP3s indeed did co-localize with BIN1 when expressed in Vero cells. The nsP3s of BEBV, BFV, CHIKV, EILV, GETV, MAYV, ONNV, RRV^{QML}, RRV^{T48}, SESV, SINV, SFV, and WHAV all showed co-localization with BIN1.

Although the proline-rich motif in alphaviruses nsP3s is often referred to as the PxPxPR motif, multiple alternative BIN1 binding motifs exist, some of which are present in the HVD of nsP3. The motif P[I/V][P/A]PPR[R/K/P][R/K][R/K] was identified having the highest affinity for BIN1 (Gotte et al., 2018). This is comparable with the results from this study; BEBV-, BFV-, CHIKV-, GETV-, and RRV-nsP3 all carry the high affinity proline-rich motif and showed a relatively high co-localization with BIN1 compared to moderate co-localization between BIN1 and nsP3 of viruses carrying other PxPxPR motifs (**Figure S2**, co-localization in ~40-70 % vs ~10-40 % of cells). Two exceptions were observed as the nsP3s of WHAV and SFV both carry the high-affinity motif, but co-localization with BIN1 was relatively poor (**Figure S2**, in ~10-20 % of cells). However, in immunoprecipitation experiments SFV nsP3 does interact directly with BIN1 and this interaction is also required for effective SFV RNA

replication (Neuvonen et al., 2011). Many of the cellular functions attributed to BIN1 stem from its ability to remodel cellular membranes (Habermann, 2004; Wu et al., 2014). Alphaviruses replicate in cytoplasmic viral factories or spherules, which are constructed from membrane invaginations (Froshauer et al., 1988). It has been hypothesized that BIN1 and related proteins help form or stabilize the membrane structures required for alphavirus RNA replication (Neuvonen et al., 2011).

Interestingly, the HVD of nsP3s of EEEV, VEEV and WEEV did not co-localize with BIN1 despite that all these viruses contained proline-rich BIN1 binding motifs (**Figure 2, S1 and S2**). Similarly, immunoprecipitation of EEEV-nsP3 and VEEV-nsP3 did not co-precipitate BIN1 whereas the nsP3s of CHIKV and SINV did (Frolov et al., 2017). Why the interaction between BIN1 and V/W/EEV-nsP3 is absent despite the presence of BIN1 binding sites remains elusive. Perhaps the proposed interaction between BIN1 and the nsP3s of V/W/EEEV is short lived and only exists during a specific stage of virus replication.

References

Aaskov, J., Jones, A., Choi, W., Lowry, K., and Stewart, E. (2011). Lineage replacement accompanying duplication and rapid fixation of an RNA element in the nsP3 gene in a species of alphavirus. *Virology* 410(2)**,** 353-359. doi: 10.1016/j.virol.2010.11.025.

Frolov, I., Kim, D.Y., Akhrymuk, M., Mobley, J.A., and Frolova, E.I. (2017). Hypervariable Domain of Eastern Equine Encephalitis Virus nsP3 Redundantly Utilizes Multiple Cellular Proteins for Replication Complex Assembly. *Journal of Virology* 91(14)**,** 1-22. doi: 10.1128/jvi.00371-17.

Froshauer, S., Kartenbeck, J., and Helenius, A. (1988). Alphavirus RNA replicase is located on the cytoplasmic surface of endosomes and lysosomes. *J Cell Biol* 107(6 Pt 1)**,** 2075-2086. doi: 10.1083/jcb.107.6.2075.

Gotte, B., Liu, L., and McInerney, G.M. (2018). The Enigmatic Alphavirus Non-Structural Protein 3 (nsP3) Revealing Its Secrets at Last. *Viruses* 10(3). doi: 10.3390/v10030105.

Habermann, B. (2004). The BAR-domain family of proteins: a case of bending and binding? *EMBO Rep* 5(3)**,** 250-255. doi: 10.1038/sj.embor.7400105.

Neuvonen, M., Kazlauskas, A., Martikainen, M., Hinkkanen, A., Ahola, T., and Saksela, K. (2011). SH3 domain-mediated recruitment of host cell amphiphysins by alphavirus nsP3 promotes viral RNA replication. *PLoS Pathog* 7(11)**,** e1002383. doi: 10.1371/journal.ppat.1002383.

Wu, T., Shi, Z., and Baumgart, T. (2014). Mutations in BIN1 associated with centronuclear myopathy disrupt membrane remodeling by affecting protein density and oligomerization. *PLoS One* 9(4)**,** e93060. doi: 10.1371/journal.pone.0093060.

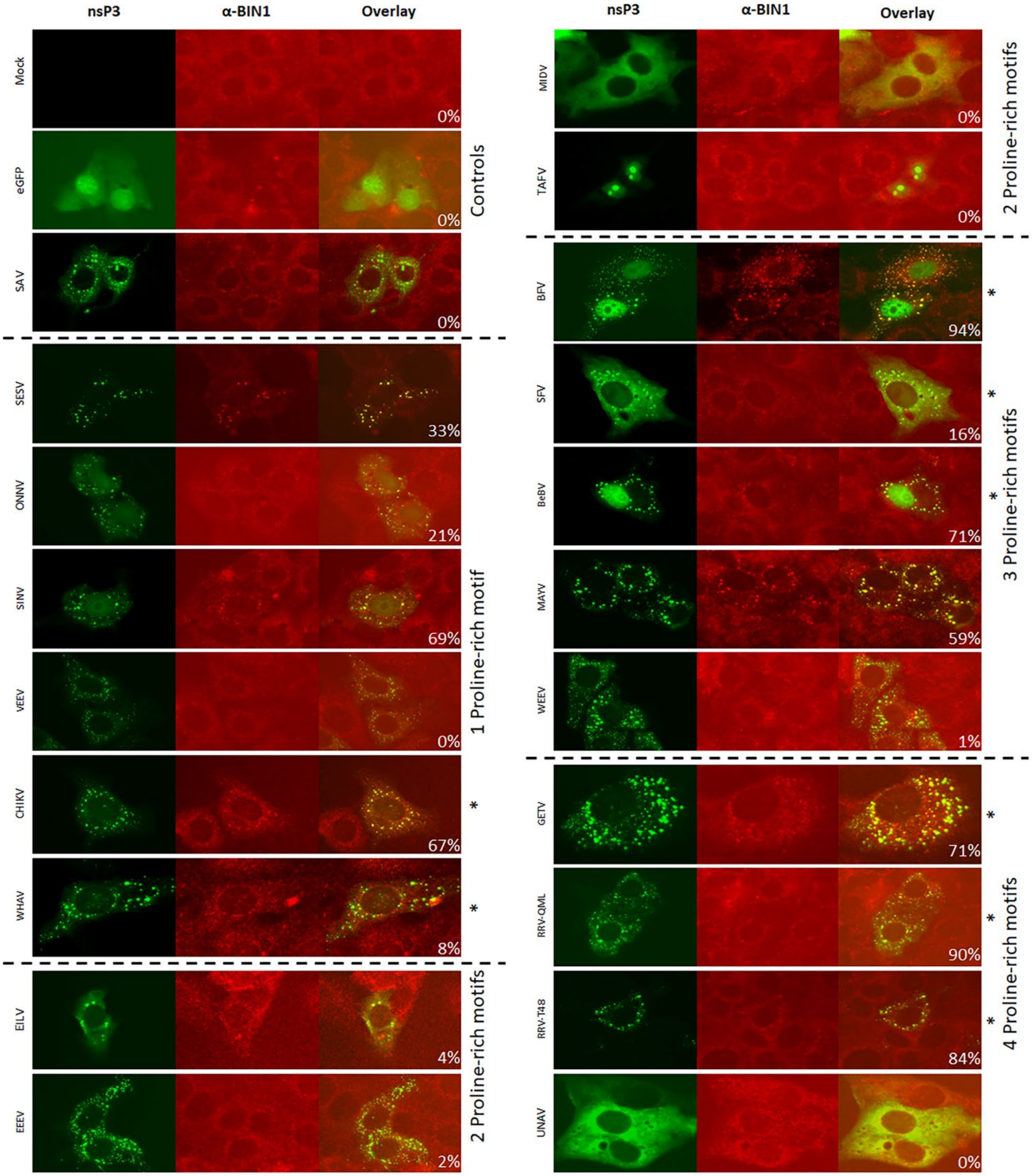

Figure S1. Alphavirus nsP3 co-localization with BIN1. Vero cells were transfected with alphavirus nsP3-eGFP, stained with antibodies for BIN1 and visualized by immunofluorescence. Green indicates localization of nsP3, red represents BIN1. NsP3-BIN1 co-localization is visualized in yellow in the overlay. The percentages in the overlay images represent co-localization percentage of all counted cells. An asterisk indicates the presence of the high affinity proline-rich motif; P[I/V][P/A]PPR[R/K/P][R/K][R/K].

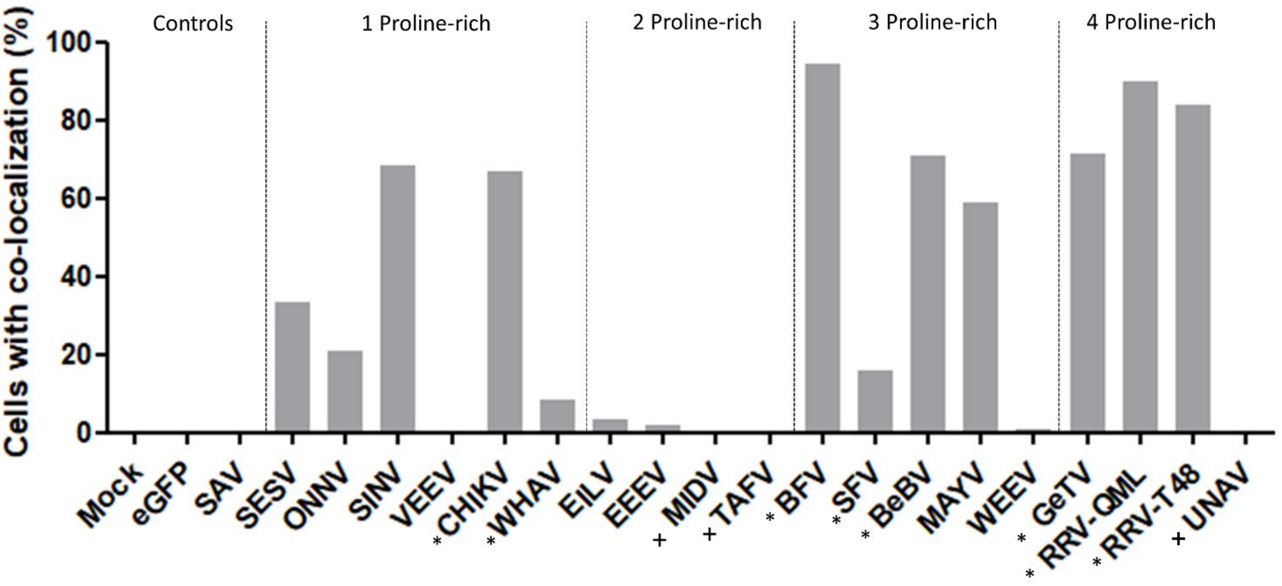

Figure S2. NsP3-BIN1 co-localization percentages. Vero cells with co-localization of nsP3 and BIN1 are represented as co-localization percentages. Bars represent the mean co-localization pattern of 30-50 transfected Vero cells. A distinction is made for alphaviruses with one, two, three or four proline rich motifs (PxPxPR). An asterisk indicates the presence of the high affinity proline-rich motif P[I/V][P/A]PPR[R/K/P][R/K][R/K]. In abscene of cytoplasmic granules, no co-localization could be determined, these nsP3-eGFPs are indicated with an open circle.

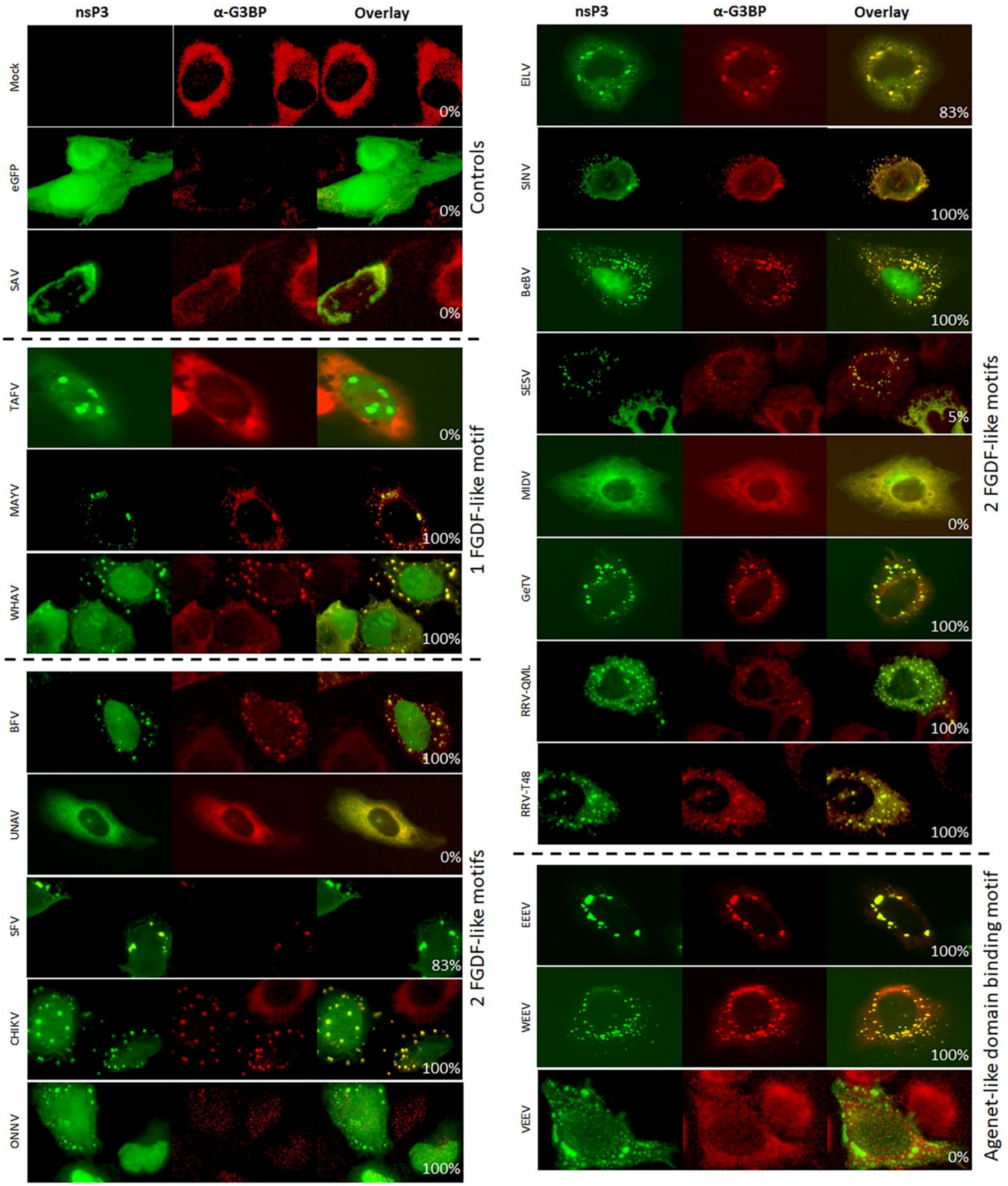
nsP3
α-G3BP
Overlay
Mock
0%
eGFP
0%
SAV
0%
Controls
TAFV
0%
MAYV
100%
WHAV
100%
1 FGDF-like motif
BFV
100%
UNAV
0%
SFV
83%
CHIKV
100%
ONNV
100%
2 FGDF-like motifs
EILV
83%
SINV
100%
BeBV
100%
SESV
5%
MIDV
0%
GeTV
100%
RRV-QML
100%
RRV-T48
100%
2 FGDF-like motifs
EEEV
100%
WEEV
100%
VEEV
0%
Agenet-like domain binding motif

Supp Figure S3. Alphavirus nsP3 co-localization with G3BP in U2OS cells. U2OS cells were transfected with alphavirus nsP3-eGFP, stained with antibodies for G3BP and visualized by immunofluorescence. Green indicates localization of nsP3, red represents G3BP. NsP3-G3BP co-localization is visualized in yellow in the overlay. The percentages in the overlay images represent co-localization percentage of all counted cells. A distinction is made for alphaviruses with one or two FGDF-like motifs or the presence of one or two Agenet-like domain binding motifs.

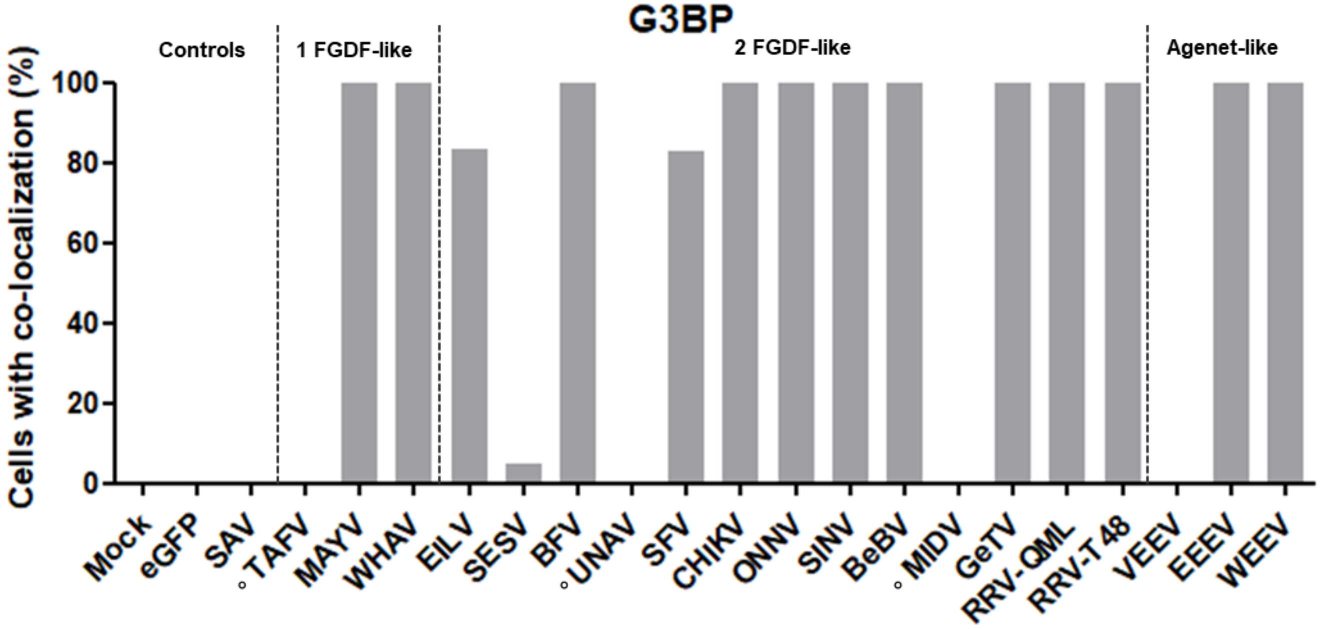

Supp. Figure S4. NsP3-G3BP co-localization percentages in U2OS cells. Cells with co-localization of nsP3 and G3BP are represented as co-localization percentages. Bars represent the mean co-localization pattern of 30-50 transfected U2OS cells A distinction is made for alphaviruses with one or two FGDF-like motifs or the presence of one or two Agenet-like domain binding motifs. In absence of cytoplasmic granules, no co-localization could be determined, these nsP3-eGFPs are indicated with an °.

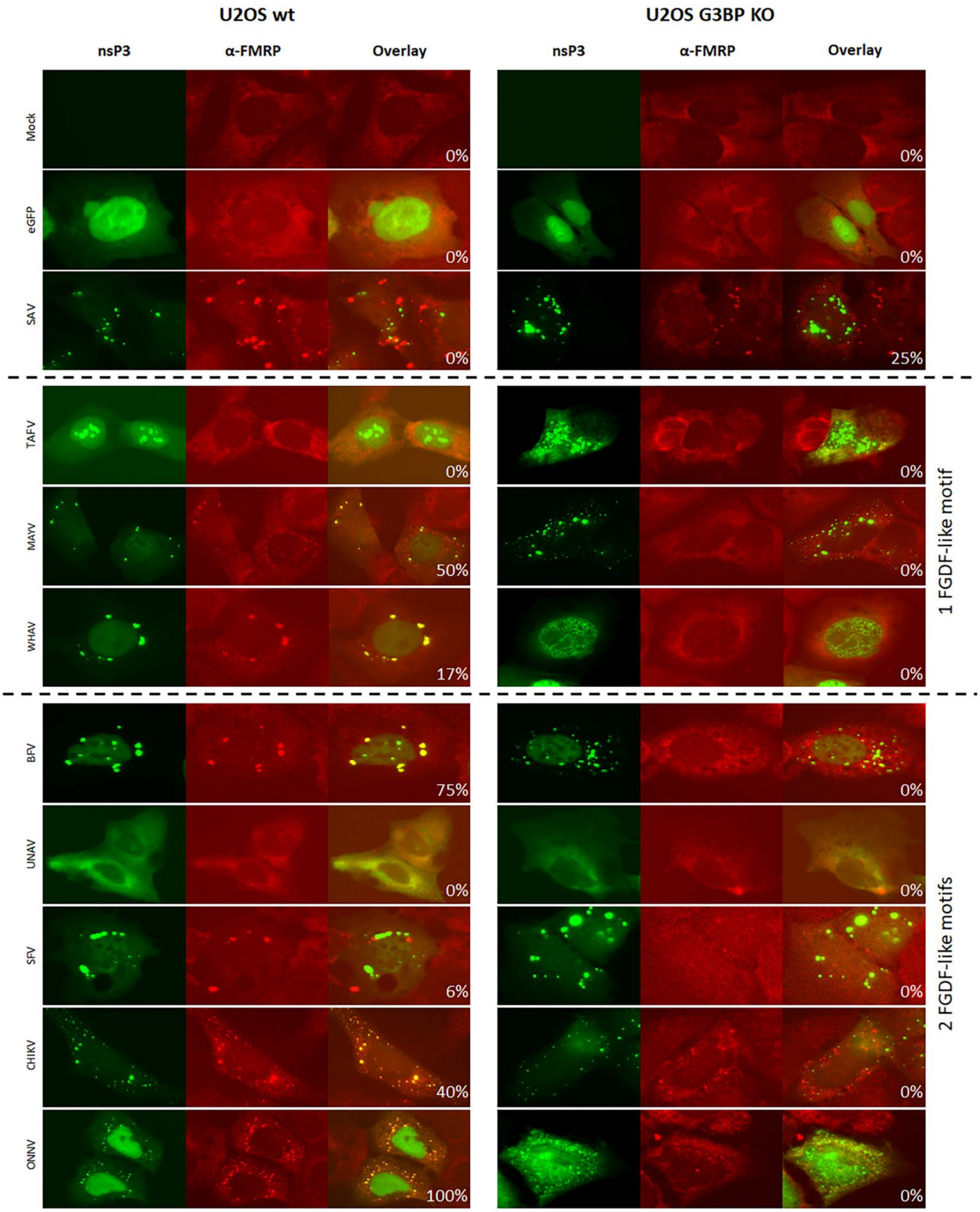
U2OS wt
U2OS G3BP KO
nsP3
α-FMRP
Overlay
nsP3
α-FMRP
Overlay
Mock
eGFP
SAV
TAFV
MAYV
WHAV
BFV
UNAV
SFV
CHIKV
ONNV
0%
0%
0%
0%
0%
25%
0%
0%
50%
0%
17%
0%
75%
0%
0%
0%
6%
0%
40%
0%
100%
0%
1 FGDF-like motif
2 FGDF-like motifs

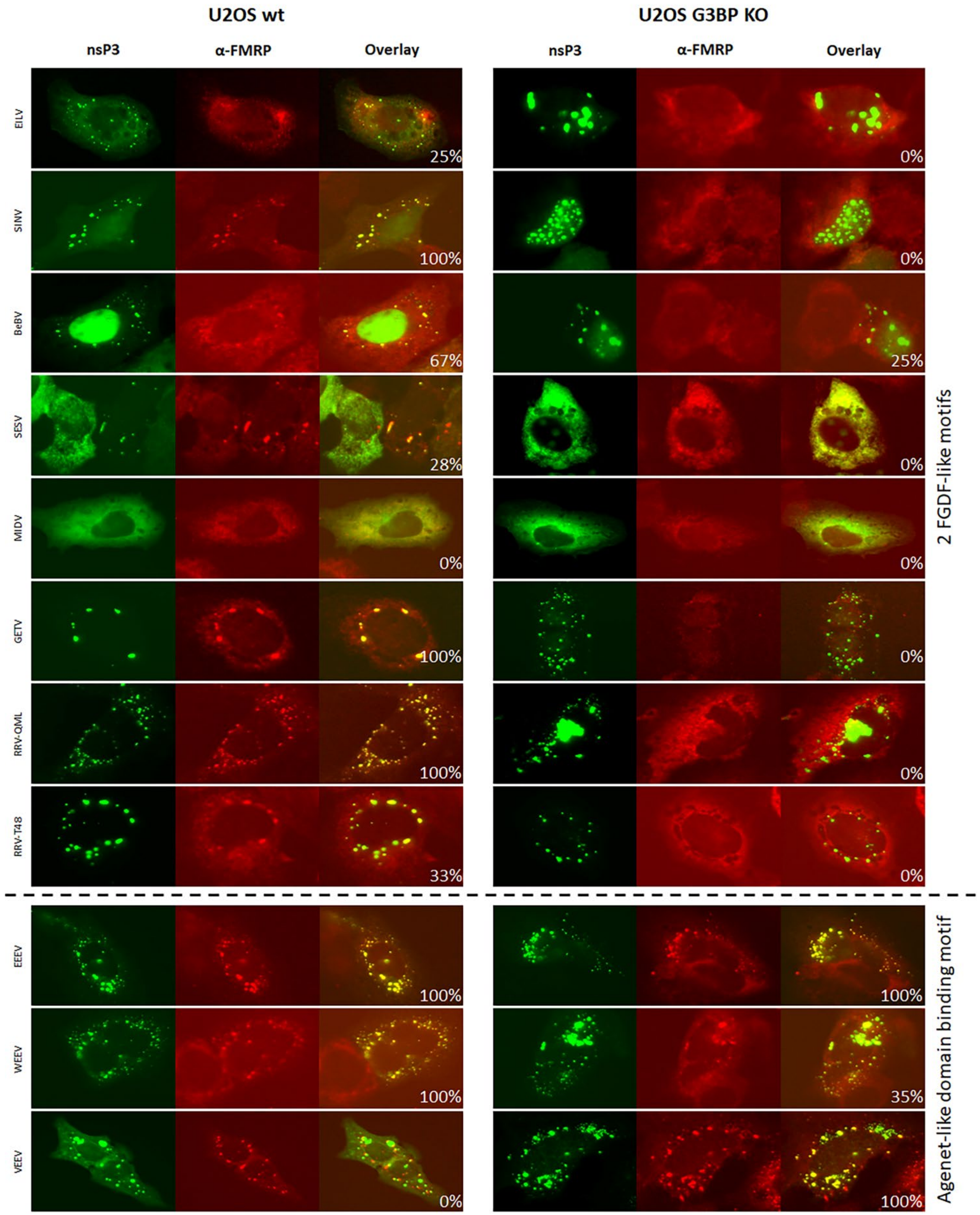
U2OS wt
U2OS G3BP KO
nsP3
α-FMRP
Overlay
nsP3
α-FMRP
Overlay
EILV
25%
0%
SINV
100%
0%
BeBV
67%
25%
SESV
28%
0%
MIDV
0%
0%
GETV
100%
0%
RRV-QML
100%
0%
RRV-T48
33%
0%
2 FGDF-like motifs
EEEV
100%
100%
WEEV
100%
35%
VEEV
0%
100%
Agenet-like domain binding motif

Supp. Figure S5. Alphavirus nsP3 co-localization with FMRP1 in U2OS wt and U2OS G3BP KO cells. U2OS wt and U2OS G3BP KO cells were transfected with alphavirus nsP3-eGFP, stained with antibodies for FMRP1 and visualized by immunofluorescence. Green indicates localization of nsP3, red represents FMRP1. NsP3-FMRP1 co-localization is visualized in yellow in the overlay. The percentages in the overlay images represent co-localization percentage of all counted cells. A distinction is made for alphaviruses with one or two FGDF-like motifs or the presence of one or two Agenet-like domain binding motifs.

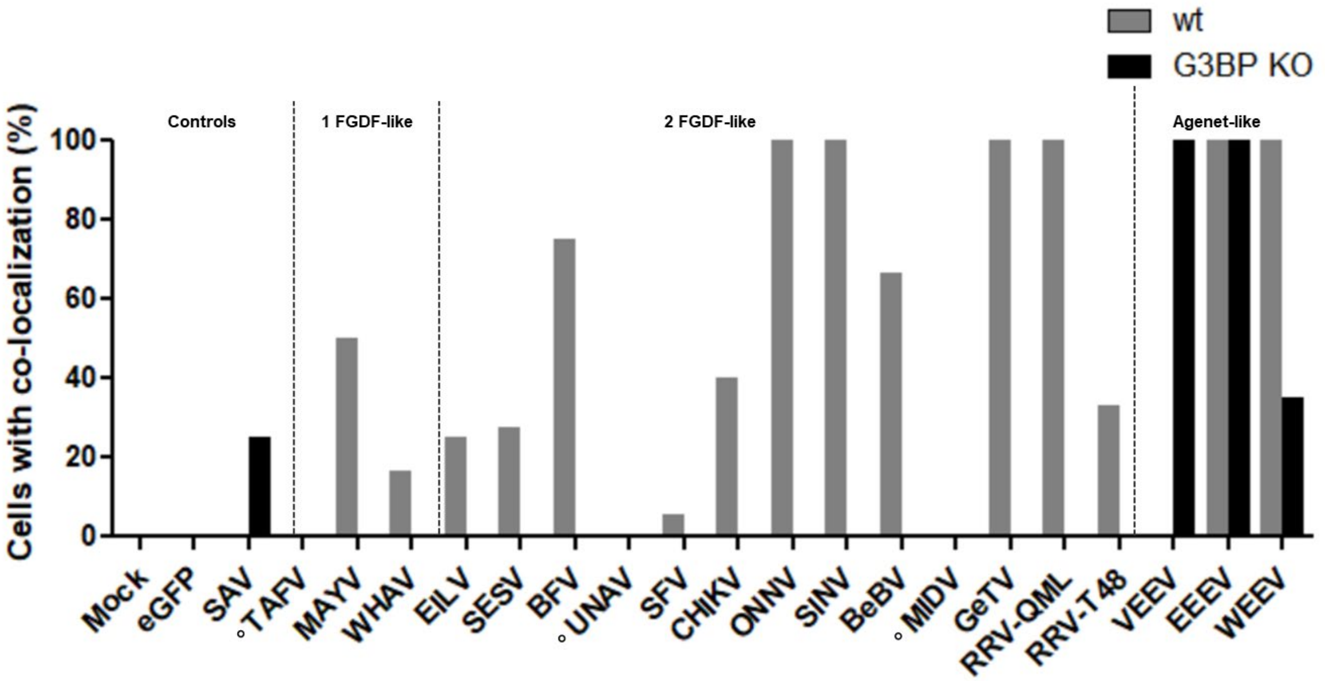

Supp. Figure S6. NsP3-FMRP1 co-localization percentages in U2OS wt and U2OS G3BP KO cells. Cells with co-localization of nsP3 and FMRP1 are represented as co-localization percentages. Bars represent the mean co-localization pattern of 20-80 transfected U2OS wt and HeLa G3BP KO cells. A distinction is made for alphaviruses with one or two FGDF-like motifs or the presence of one or two Agenet-like domain binding motifs. In absence of cytoplasmic granules, no co-localization could be determined, these nsP3-eGFPs are indicated with an °.

NsP3 gene sequences of the 20 alphaviruses used in this study.

```
> SAV

atggcaccgggttacagagtcctcaacaggaacatcatcactgccgaagaggaagtcttggttaacgccgctaacagtaac
ggcagacccggcgatggtgtgtgtggtgcgctctacggcgcgttcggggacgcattccccaacggtgcgatcggcgcggga
aacgcggtcttggtccgaggactcgaggccaccatcatacacgcagccggagctgacttcagagaagtcgacgaagaaacc
ggcgcgcgacagctaagagcagcataccgtgcggcggctaacctagtcactgccaacggtatcaccagtgctgccatcccc
ttgctgagtacacacatcttctccaacggtcgaaacagactggaacagtccttcggcgcattggtggaggcgttcgacacg
acagaatgcgacgtcaccatctattgcttggccaacaacatggccgtaaggattcagcaactgatcgacgatcacgcccgc
gaagagttcgacgaggaggtggttgtagaagaggaagaggaacatgaagctgatgcgatgagtgatacggagacgctgtcc
agtttcggtgacgaaacggtgtgggtgcccaaacatagcactctagctggaaggccaggatatagtgcctcttacggcgac
cgcagatcccttttgtcggcacgaagttccaccgcgccgcagttgctatgtcgtcaattgaagcggcttggcctaagacc
aaagaagccaacgccaaactcatcgagtacatacgagggcaacacctcgtcgacgtcctaaagagctgtccggtcaacgac
atacctgtaggcagaccgccttctagcctgccctgcggctgtatttatgccatgaccccggaacgggtcacagtgctgaag
caaaggccgcaggaaggttttgtggtatgcagcgcattcaaattgccgctcactaacatccaagatgtcaccaaagtggag
tgcacggtgagagcacctgcagaagaacctagacccgtgcgctatctgcaagagagacgcccagtccaggccgccgtgggg
caacctaggccggcaactgtggctgccagagtcgccgcgagtcatgcagccagtgggaccagcacagccacgagccgccac
acacccgcgccgggctcggtgcgggtgcgcctgctgccgccaagagacggtacggtatcccgcagctctcgcacgggttcg
cagtccagcgtcacctcatcagcgggatccatactgccagtgccccgaagggcgccagttacgccagcggcatcattggcg
ggcagcgtccacggccatagtgtacgcagcgcccctgccatgagggccgccagcacaggtgccagaagcgtgcgcagtgtc
cagtccggttcagccgggcatagaactgacgtcgccagcgtcgccggctcagcggggctgcctagagggctaacacgggac
cagttcggcgccgtgagagctagggcccgcagggacttagagctggagggatcagagcatggcagtcagactagcttccgt
tccggctcgctgatggtggagagcaccgctagtggctatagccaatgttctgacgatcaggacacgggctccgaaccatca
agccgcggcgcctctgtgaggacgcggcgcagagggcaacgagacggccctggagggcattga


> SESV

atggccccgaactatcgtgttgtcagagccgacatagctaaaacgacaactgaagcagtggtcaacgccgccaacccccctg
ggagtgccgggcgcaggtgtgtgtggggctatcgctagacagtggcctaaaggatttccggcaggtaaaatgcaggttggt
gagtgtaaagcggtagtaacagatgatatcaccatactgcatactgttggccctgacttccgtaagactggggaaaaggag
ggcgacgagctgctggcgatggcctaccagaattgcgctgcagaggcgtcctccttagggttccacaccttagctatccca
ctattatccaccggcatatattcagctggagtagatagattagatcagtcgctaaaacatttgctgaatgcttttgatgag
accaacatagaggttcatatatattgtagagataaaacatgggagtctcgcattttgagagtgattagtacgcgggacact
gtggaggaattggtagaggaaaatgtggaactgtcagatgaaatcgtaagagtccacccagctagctcattggagggaagg
gaagggtatagctccaccactggatcaatctactcattttgcctggtaccaagtttcatcagacagctgtggacgtggcg
gagatcaaagccctgtggccaaaggaaccagaggcaaatgagcagatatgtaattacattctcggggatagcatgaaccag
atacgagacaaatgcccggtggaagatataggcactacatcccctccatgtacggtaccatgtttatgtccttatgccatg
actgcagaaagagtgtacagagtgagagctacagcgcctaagagcttcattgtgtgttcatcattccagctaccaaaatat
cgtattactggagtgcaacgggtgccctgtacgcgacctttaaacaagttaggtcctctggagcaagtaaagaagtggca
gaggaggtttcgctgagacctgcgagtagcttctcaggccgctacggagaaagactgacggttaaggcggatgtctactcc
ttgatagacagtcgctgggattccattgactccgtcaagttgccgagtttggatactgatagtaccacgctttctgagact
cctccaccgcgctatgatacacctgcggttgcgaccacaccgccagtacccccgccgcgtctgaagaagctagcacggcaa
caagcgctgtcgcaaatttcttttggtgattttgaaccaggagagcccgatagactagctgccgaaagcagggcatctaga
gaggccccgtctattacatttggagatttccaggaaaatgaggtggacagtatagttagcgcgccgccgattactaagtca
caacgtaggcgtaggagacgcaaggcgcgaagatccgcgtctaggcagcctttgctggactccccgccattatga


> SFV
```

atggcaccatcctacagagttaagagagcagacatagccacgtgcacagaagcggctgtggttaacgcagctaacgcccgt
ggaactgtaggggatggcgtatgcagggccgtggcgaagaaatggccgtcagcctttaagggagcagcaacaccagtgggc
acaattaaaacagtcatgtgcggctcgtaccccgtcatccacgctgtagcgcctaatttctctgccacgactgaagcggaa
ggggaccgcgaattggccgctgtctaccgggcagtggccgccgaagtaaacagactgtcactgagcagcgtagccatcccg
ctgctgtccacaggagtgttcagcggcggaagagataggctgcagcaatccctcaaccatctattcacagcaatggacgcc
acggacgctgacgtgaccatctactgcagagacaaaagttgggagaagaaaatccaggaagccattgacatgaggacggct
gtggagttgctcaatgatgacgtggagctgaccacagacttggtgagagtgcacccggacagcagcctggtgggtcgtaag
ggctacagtaccactgacgggtcgctgtactcgtactttgaaggtacgaaattcaaccaggctgctattgatatggcagag
atactgacgttgtggcccagactgcaagaggcaaacgaacagatatgcctatacgcgctgggcgaaacaatggacaacatc
agatccaaatgtccggtgaacgattccgattcatcaacacctcccaggacagtgccctgcctgtgccgctacgcaatgaca
gcagaacggatcgcccgccttaggtcacaccaagttaaaagcatggtggtttgctcatcttttcccctcccgaaataccat
gtagatggggtgcagaaggtaaagtgcgagaaggttctcctgttcgacccgacggtaccttcagtggttagtccgcggaag
tatgccgcatctacgacggaccactcagatcggtcgttacgagggtttgacttggactggaccaccgactcgtcttccact
gccagcgataccatgtcgctacccagtttgcagtcgtgtgacatcgactcgatctacgagccaatggctcccatagtagtg
acggctgacgtacaccctgaacccgcaggcatcgcggacctggcggcagatgtgcaccctgaacccgcagaccatgtggac
ctcgagaacccgattcctccaccgcgcccgaagagagctgcataccttgcctcccgcgcggcggagcgaccggtgccggcg
ccgagaaagccgacgcctgccccaaggactgcgtttaggaacaagctgcctttgacgttcggcgactttgacgagcacgag
gtcgatgcgttggcctccgggattactttcggagacttcgacgacgtcctgcgactaggccgcgcgggtgcatga

> SINV

gcctcataccgcaccaaaagggagaatattgctgactgtcaagaggaagcagttgtcaacgcagccaatccgctgggtaga
ccaggcgaaggagtctgccgtgccatctataaacgttggccgaccagttttaccgattcagccacggagacaggcaccgca
agaatgactgtgtgcctaggaaagaaagtgatccacgcggtcggccctgatttccggaagcacccagaagcagaagccttg
aaattgctacaaaacgcctaccatgcagtggcagacttagtaaatgaacataacatcaagtctgtcgccattccactgcta
tctacaggcatttacgcagccggaaaagaccgccttgaagtatcacttaactgcttgacaaccgcgctagacagaactgac
gcggacgtaaccatctattgcctggataagaagtggaaggaaagaatcgacgcggcactccaacttaaggagtctgtaaca
gagctgaaggatgaagatatggagatcgacgatgagttagtatggattcatccagacagttgcttgaagggaagaaaggga
ttcagtactacaaaaggaaaattgtattcgtacttcgaaggcaccaaattccatcaagcagcaaaagacatggcggagata
aaggtcctgttccctaatgaccaggaaagtaatgaacaactgtgtgcctacatattgggtgagaccatggaagcaatccgc
gaaaagtgcccggtcgaccataacccgtcgtctagcccgcccaaaacgttgccgtgcctttgcatgtatgccatgacgcca
gaaagggtccacagacttagaagcaataacgtcaaagaagttacagtatgctcctccacccccttcctaagcacaaaatt
aagaatgttcagaaggttcagtgcacgaaagtagtcctgtttaatccgcacactcccgcattcgttcccgcccgtaagtac
atagaagtgccagaacagcctaccgctcctcctgcacaggccgaggaggcccccgaagttgtagcgacaccgtcaccatct
acagctgataacacctcgcttgatgtcacagacatctcactggatatggatgacagtagcgaaggctcactttttttcgagc
tttagcggatcggacaactctattactagtatggacagttggtcgtcaggacctagttcactagagatagtagaccgaagg
caggtggtggtggctgacgttcatgccgtccaagagcctgcccctattccaccgccaaggctaaagaagatggcccgcctg
gcagcggcaagaaaagagcccactccaccggcaagcaatagctctgagtccctccacctctcttttggtggggtatccatg
tccctcggatcaattttcgacggagagacggcccgccaggcagcggtacaacccctggcaacaggccccacggatgtgcct
atgtctttcggatcgttttccgacggagagattgatgagctgagccgcagagtaactgagtccgaacccgtcctgtttgga
tcatttgaaccgggcgaagtgaactcaattatatcgtcccgatcagccgtatcttttcctctacgcaagcagagacgtaga
cgcaggagcaggaggactgaatat

> BeBV

atggcaccctcgtacagggttaagcgtgcagacatcgctactagcgaggaagaggcagttgtaaacgccgccaacgcaaaa
ggtagaccgggagacggagtgtgcagagccatccaccgaaagtggcctgaggccttcgtgggcgctgccacggcgaccggc
acggcaaaaaccataaaggtcggacagacatacatcatccatgccgtcggaccgaacttttcctcgacccaagaacaggag
ggtgataagttgctggccggagcgtaccgagcggtagccgaagaagtaattaaatacgggtgccgcagcgtcgccattccg
ctgctgtctacgggcatttatggcggtggtaaagaccgaatgtaccagtcgttgaaccatctttttaccgcgttggatgcg
accgacgcagatgtggtgatctattgcagggacaagacgtgggagacgaagatccaggaggcgatagatcgcagactggcg
gtggagctagtgtccgatgaaatggaactgcagacagacttagtccgcgtccatcccgatagcagcttagtgggacggcgg
ggttacagcactactgacggtaagctgtattcatacctagagggcaccaagttccatcaatgtgccgtcgatatggcagag
attctggtattatggccgaacaccagggaggcgaatgagcaaattgcattatatgccctaggagaaagtatggacactata

agatctaggtgcccagtagatgacaatgattcgtcttcaccaccgcggacggtgccatgtctgtgtaggtatgctatgaca
gccgagagggtgaccagattacgcatgcaccatactaagtcgttcacagtctgttcttcattccccttgcctaaatacaac
gtggaaggggttcagagggtgaaatgcgagaaggttctactgtttgatccgacggttccttcgctggtgagccctaggaag
tacgtgtgtaacaccactatacaggccgatgatctctcctcgatcacggagtgttccttatcgtcacgtagaccatctgtc
tcaatatctgtctcctccatttctacaacagatttcatgccgcgaaacacttccgtggacaacatactacgggtcattgcc
gagattcaccctgtcccaaccgaggtacagacgctgcctgtgcccgagcaaggggatgtcccctgcggcacattaccggtg
gagcatcaagcacccgtgccccctcctagaccgaagcgcgccagggcattggcggctgccaggataccaccagttccggcg
ccgagacattcaaaggcacgtcccgttccggccccgaggaccattttcagaaccagtaggcccgttgtccgagcagccgtt
gaactaccatggaaaatacaggttgtgcctgggctgactttcggcgatttgccggagccctcgagcacaccagccgttgag
ctgccatgggagccggaggagtcgtcaggactatcatttggcgacttcgggacgttctga

> BFV

atggcaccggcgtacagagtcaaacgtggagacatttcgaacgccccagaggatgcagtggtcaatgcagcaaaccaacag
ggagtgaagggtgctggagtttgcggtgcaatttaccgtaagtggccggacgctttcggtgatgtcgctactccaaccgga
acagcagtttcgaaatccgtccaagataaattggtgatccacgctgtcggcccgaatttctcaaaatgttcagaagaggaa
ggggacagagacctagcatctgcttacagagctgcagcagaaatagtgatggataaaaaaattacaacagtggccgtcccc
ttactctccaccggcatttatgccggaggaaaaaacagagtagaacagtcactcaaccatctcttcacggcattcgacaat
actgatgcagatgtgaccatatattgcatggacaaaacatgggaaaagaagattaaggaggcaatcgatcaccggacttcg
gttgagatggtgcaggatgacgtgcagttggaggaggaactggtacgagtacaccctttgagtagtttagcaggtaggaag
ggttacagtacggacagcggccgagtgttttcctacctggaaggtaccaaattccatcagactgcggtggacatagccgaa
atgcaagtgctgtggcccgccctcaaagagtctaatgagcaaatagtggcatacaccttaggagaatcaatggaccagata
cgtggcaagtgcccgacagaagatactgacgcctccacacctccacggactgtgccgtgcctctgtcgatacgccatgaca
ccagagagagtgtaccgacttaaatgcacgaacactacccaatttacggtttgctcatcttttgagttgccaaagtatcac
attcagggagtgcagagagtaaaatgtgaaagaatcatcatcttagatcccactgttccaccaacttacaaacggccatgc
atcagacggtacccctccacaatctcttgtaactcctctgaggactccaggagcttgtctactttctctgtcagctccgac
tcctcgattggttctctgccggtcggagacacgagacccattccagccccgaggaccattttcagacccgtccctgccccg
agagcacccgtgctcagaaccacaccgcctcctaaaccaccgcgcacattcaccgtgcgtgcagaagtgcaccaagcaccc
cctacacctgtacctccacccagaccgaagagggctgcaaagttggctcgtgagatgcaccccgggttcaccttcggggac
ttcggagagcacgaggttgaggagcttacggcctctcccttaaccttcggagattttgctgaaggagagatccaggggatg
ggagtggagtttgaatga

> CHIKV

atggcaccgtcgtaccgggttaaacgcatggacatcgcaaagaacgatgaagagtgtgtagtcaacgccgccaaccctcgt
gggctaccaggcgatggcgtctgtaaagcagtatacaaaaaatggccggagtccttcaagaacagtgcaacaccagtggga
accgcaaagacagtcatgtgcggtacatacccggtaatccatgcagtaggacctaatttctcaaattactctgagtccgaa
ggagaccgggaattggcagctgcttaccgagaagtcgctaaggaggtgactagactaggagtaaacagcgtagctataccg
ctcctttccaccggtgtgtactctggagggaaagacaggctgactcagtcactaaaccaccttttacagcattagactca
actgatgcagatgtggttatctactgccgcgacaaggagtgggagaagaaaatagctgaggccatacaaatgaggacccaa
gtggaattactagacgaacacatctctgtagactgcgatatcatccgagtgcaccctgacagcagtttggcaggtagaaaa
gggtacagcactacagaaggttcactgtactcctacttggaagggacacggttccatcagacggcagtggacatggcagaa
gtatacaccatgtggccaaagcagacggaggctaatgaacaagtttgcttgtacgcattggggggaaagtatagaatcaatc
aggcaaaagtgcccagtggatgacgcagatgcatcgtcgcccccaaaaaccgtcccgtgcctctgccgttatgccatgaca
cccgaacgagtcaccaggcttcgtatgaaccatgtcacaagcataatagtatgctcatcattccccccttccaaagtataaa
atagaaggagtgcagaaagtcaagtgttctaaagtgatgctgttcgaccataacgtgccatcacgcgttagtccaagggaa
tataaatcgcctcaggagaccgcacaagaagtaagttcgaccacgtcactgacgcacagccaattcgaccttagcgttgac
ggtgaggaactgcccgctccgtctgacttggaagctgacgctccgattccggaaccaacaccagacgacagagcggtactt
actttgcctcccacgattgataatttttcggctgtgtcagactgggtaatgaataccgcgccagtcgcaccacccagaaga
agacgtgggaaaaacttgaatgtcacctgcgacgagagagaagggaacgtacttcccatggctagcgttcggttcttcaga
gcggatctgcactccatcgtacaggaaacggcagagatacgcgatacggccgcgtccctccaggcgcccctgagtgtcgct
acagaaccgaatcaactgccgatctcatttggagcaccaaacgagactttccccataacgttcggggattttgatgaaggg

gagattgaaagcttgtcctctgagttactgacctttggggacttctcgccgggcgaagtggatgacctgacagacagcgac
tggtccacgtgttcagacacggacgacgaattatgactagatagggcaggtgggtga

> EEEV

atggcacctgcgtatagagtggtgcgcggtgacataacaaagagcaatgatgaggttattgttaacgcggcgaacaacaaa
gggcaacccggtggcggtgtgtgtggcgccctttacaggaagtggcctggagcttttgataagcagccggtagcaactggt
aaagcgcacctcgtcaagcattctccgaacgtcatccatgccgttggccctaatttttctcggctatcagaaaacgaagga
gaccagaaattgtctgaagtgtacatggacattgccagaattatcaacaacgagaggtttactaaagtctccattccgttg
ttatctaccggcatctatgcaggtggtaaggacagggttatgcaatcgctgaaccatttatttacagccatggatactacc
gacgcagacattaccatttactgtctagataagcaatgggagtcaagaataaaggaagctatcacgcggaaggaaagcgtt
gaagagcttactgaggatgacagaccagttgacattgaactggtacgggtgcacccgttgagcagcttggcaggtagacct
ggttattcaaccaccgagggcaaggtgtattcgtacctagaggggactaggtttcatcaaactgccaaagacatagctgaa
atttacgctatgtggcctaacaagcaagaagcaaacgagcagatttgcttatacgtgctgggagagagtatgaacagcatc
cgctctaagtgtccagttgaagagtcggaggcctcttccccccctcacaccatcccgtgtctgtgcaactatgcaatgact
gcggagcgagtttacagattacgtatggcaaagaatgaacaattcgcagtttgttcgtcctttcagttaccgaaatacagg
attacaggggttcagaaaattcaatgcagcaaacctgtgatattctctggcactgtaccccggccatacatccaagaaaa
ttcgcatctgtgacagtggaagacactccggtggtccaacctgaaaggttggtgcctaggcgacctgcaccgcctgtgccc
gtacctgcaagaatccccagccctccatgtacatcgaccaacggatcgacgaccagtatacaatcactgagggaggatcaa
agcgcatctgcctctagcggagctgaaatctccgtagaccaggtttcgctatggagcatacccagcgctactgggttcgat
gtgcgtacctcctcatcgttgagtctagagcagccaacctttccgacaatggttgtcgaagctgagattcacgccagtcaa
ggatcactgtggagcatacccagtatcaccggatctgaaatccgtgctccgtcacctccaagtcaggatagtagaccttcc
accccatctgcaagtggttcacacacgtccgtggacttaatcacgtttgacagcgttgcagagattttggaggatttcagt
cgttcgccgtttcaattttgtctgaaatcaaacctattcccgcacctcgtacccgagttaataacatgagccgcagcgca
gacacgatcaaaccagttccaaagccgcgtaaatgccaggtgaagtacacgcagccacctggcgtcgccagggccatatcg
gcagcggaatttgacgagtttgtgcggaggcactcgaattga

> EILV

atggccccatcatactcggtgatcagaggcgacataaccgcgactaactcccacgccattgtcgtccctgtcacgccggag
cgaaaagacggcgtgtatcgcgcttgtagcaagaaatgggccccctacctcgcctggagtggaccgaaggtgccaccttg
ttctcgcccggttcaccagccactctgcaagtatgtgtaccctcgctccagaatacggacactacatcaacccagcaagcc
taccgcgccatcgccaaagttgtcgtcgacgagcagattccgtcactatctctacccgtcctcaccatgaagaagaccggc
acagcagacaccgtatcagaatccttgaaccacctagttaccgctctggaccaaaccgatgcaaatgtaactatttactgt
ctcgacaaaagcaggctcataaaaatcaaggaagtaattgcacgcaaggaagccgtcaccgagcttatcgacgacgaccta
gaaatcgacgaggaactgacatgggtccaccccgatagctgcctacgcaaccgcaccggttttagcaccgacaaaggaaaa
ctgtactcatatctggaagggaccaagttccaccagatggccaaggacttcgcagagattaggtcactattccctgacgag
atggaagctaacgaacacatctgctcactcatcttaggggaaacgatagatggcatccgagaacgctgtccagtgacagac
aatccgccatcatcaccgcccaagactgtaccctgcttgtgcatgtacgccatgaccccagaacgcgccctacggctcaag
agcaattctgtcacccaaatcacagtctgctcgtccttcgttctcaagaagcaccacatcaaaggggtacagaagatccaa
tgcacggcacctatgttattcaacccgacaccattaacttccaggacggtccgcactccgccacaagtctcagcacgagcc
gcactcgatcttcctcccgttgcacctatgccttctgtacctgcaccggttagcctgacgcctacgaggcgtgcaccacca
ccgccccttaccaaacgacccgttgtcgtacgtccgtcgacgcctccaccgccgccaccagtacgccagacaccaacgcca
gtgctcgcgccacggactggttctacggcagcacccactccgacgccacgcctctcgttatctacggaccagccatccgta
gacatttcgttcggagacttttcccccgcagaaacgatgtctttgatgctgtcgtcccctggctctgacaccgccagtatc
accttcggtgacttcgacgaggacgaggtagaatctatagtaggacgggaatat

> GETV

atggcaccatcatacagggtccgccgcgcagatatatcaggacacagtgaggaagcggtcgtaaatgctgccaatgccaaa
ggtaccgtgagcgacggagtgtgcagggcggttgctaagaagtggccatcatctttcaaagggggctgcaactccagtcggc
acagccaaaatgatccgcgcagatggcatgaccgtaatccacgcagtgggaccaaacttctccaccgtaacagaagccgaa

ggggacagagagctagcggccgcgtatcgagctgtggctagcataattagtaccaacaacataaagagcgtcgcagtaccg
ctgctgtccacaggcaccttctccggcggtaaggacagagtgacgcagtccttgaaccacttattcacggcactggacgca
accgacgcagacgtggttatctactgcagagataaaaactgggaaaagaagattcaggaagccatcgacaggcggacggca
atcgagctcgtatctgaagacgtgaccttggaaaccgatctggttagagtacacccggacagttgcttagtcggcagaaat
ggttacagtgcaactgacggtaaactgtactcttaccttgagggcacgaggttccaccagacggcggtcgacatggctgaa
atatcaactttatggccaagactccaagatgctaacgagcagatctgcctatacgccctaggggagacgatggatagcata
cgcactaaatgcccagtagaggacgcagattcgtctacgccgccgaaaacggtaccgtgtctatgtcggtatgcgatgacc
gcggagcgggttgccagacttaggatgaataacaccaaaaacatcatcgtgtgctcctcctttccattaccgaagtacagg
atagaaggcgtgcagaaggtgaagtgtgaccgagtgctaatttttgaccagaccgtcccgtcactagtaagtcccagaaag
tacatacagcagccgccggaacagctggataatgtgagcctgacttctacgacgtcgacgggatccgcatggtcatttcca
tcggaaacgacctacgaaaccatggaagtcgtagccgaggtacataccgaacctccaatccctccgcctcgccgacgtaga
gcagccgtcgcccaacttagacaggatctggaagtcaccgaggagatcgagccgtacgtggcacagcaagcagaggtcatg
gtcttggagagggtcgcgacgacagacatacgcgctatcccagtcccggcacggcgggccatcacaatgccagtcccaacc
cccagggttcgtaaggtcgctactgaacctccattagaaccggaagctcctatcccggcaccaagaaagagaagaaccact
agcaccccacctccgcataaccccgaggatttcgttcccagggtacctgttgagttaccgtgggagccggaagacctagac
atccaattcggtgacttggagccacgccgccggaacaccagggaccgagatgtcagcacaggaatacagttcggtgacatc
gactttaaccagtcc

> MAYV

atggctccagtgtatgccgttaaaagggccgacattgcaaccgctattgaggacgcggtggtcaacgcggccaaccaccgc
ggacaagtgggcgacggtgtctgcagagctgtagcacggaagtggcctcaagccttccgcaacgcagcaacaccggtcgga
accgcaaaaaccgtcaagtgcgacgagacctacatcatccatgcggtggggccgaactttaacaatacatccgaggccgag
ggagatcgtgacttggcagcggcataccgagccgtggcagcagagatcaaccgactgtccataggtagtgtggcaatccca
ctgctttctacaggtatatttagtgctgggaaagacagagtgcatcaatcactctcgcatctgttggcagcaatggatacc
aytgaggcacgggtcactatctactgccgcgataaaacgtgggagcaaaagattaaaaccgtcctgcaaaaccgcagtgcc
actgaactggtgtcagatgaattacagtttgaagtcaacctgactagagtccatccggacagtagcctggtgggacgtcca
gggtacagcactaccgatgggactctgtactcctacatggaaggtactaagttccaccaggcggctcttgacatggctgag
atcacgactttgtggccaagagtccaggatgcaaatgaacatatttgtttgtatgcgctgggcgagaccatggacaatatt
cgtgccaggtgcccagtggaagacagtgattcatcgactccacccaagacggtcccatgcctatgccgatatgccatgaca
ccggagagagtcacaagactacggatgcaccacacaaaagatttcgtggtttgctcgtcttttcagctgccaaagtatcgt
atacctggcgtgcaacgagtgaagtgtgagaaagtaatgctttttgatgcagctccaccagcctccgtcagtccagtgcag
tacctgacaaaccaaagtgaaactaccataagtttgagttcattttcaattacatctgacagcagctccctcagcactttt
cctgatctggagtcagcggaagaatttgaccatgactctcagtccgtgcggccagcgctgaatgaacccgatgaccatcaa
cctacgccaacggcagaacttgccactcatcccgtaccaccgcctcgccctaaccgtgcaaggcggttggcggccgctcgt
gtgcaagtccaggtggaagtacatcaaccacctaccaaccaaccaacgaagccaatcccagcaccacgtaccagcctacgc
cccgtcccagctcctagaagatacgtgccaagaccggtggtcgagttaccctggccgctggagaccatcgatgtagagttc
ggggcaccgactgaagaggagagcgatatcactttcggtgacttctccgcctcggagtgggaaaccattagtaattcatct
tga

> MIDV

atggcaccatcatatagagtcgtaagaggcaatatcaccgactccgacgccgatgtgctagtaaatcagctgggcgtgaac
aacaaggtctgcgacggagtttgtagggccatggtcaagaagtggccttcctgcctaccaacgacaactggaaaagtaggc
gacgccgtgctgaccactgagccccgaaaaatcgtgcacgcttactgtcctaatttcggaaccagtagagaggaggttgcc
gatgccgacctagcagctgtgtatagagccgtggcgtccttggctgatgagacagtccgcacaatggccataccactcctg
tcaacggggacgttcgctggggaaaggaccgcgtgctgcagtcgttgaaccacctatttacggccctggacaccacggac
gtcgatgtaacgatatactgccgggataagtcgtgggaaaagaaaatccaagaggccattgatatgaggacggcaaccgaa
ctgctagatgacgacacaacggttatgaaagagctaaccagggtgcatcctgatagctgcctagtggggcgcagtggattc
agcacggtggacggacggctgcattcgtaccttgaaggaactaggttccaccagactgctgtcgacgtggcagaaatagcc
actctgtggccaaggagagaagaagcgaacgagcagataacacactacgtccttggcgaatccatggaggccataagaacc
aaatgcccggtggatgacaccgattcgtcggcaccaccatgcaccgtcccgtgcctttgccgctacgccatgacccccgag
cgcgtacatagattgcgcgccgcgcaggtgaagcagttcacggtctgctcctcgttcccgctgccaaagtacaagatacca

ggcgtgcagagagtggcgtgttcggctgtaatgttgtttaatcacgacgttccagcgctggtaagccctcgcaagtacagg
gaaccgagcattagcagcgagtcgtcatcctctggactgtctgtgttcgacctggacataggctctgattcagagtacgaa
ccaatggaacccgtgcaacccgaaccgctgatcgacttggcagtcgtagaggagacggcccccgtcagactagaacgggtg
gcccctgtggctgcacctcgcagagcccgcgcgacaccctttactttggagcagcgggttgtagcaccagttcctgcgccg
cgtacgatgccagtcagacccccctcgccggaagaaagcggcgaccagaacacctgaaaggatttcgttcggcgatttagat
gccgagtgcatggccatcatatatgacgacctgactttcggggacttcggcgcgggcgagttcgaacgtttaacgtcagca

> ONNV

atggctccgtcataccgtgtgaaacggatggacatcgcgaaaaacactgaggaatgcgtggtaaacgccgccaatccacgc
ggagtaccaggcgatggagtatgtaaagccgtgtatagaaaatggccagagtcattcagaaacagtgcaacaccagtgggg
actgcaaagacaatcatgtgcggccaataccccgtcatccacgcagtaggtcctaacttctcaaactattctgaggctgaa
ggggatagggaattggcttcagcgtatagagaagtggcgaaagaagtgtctaggctaggagtgagcagtgtagccatccct
ttgctttcaaccggtgtgtactcaggaggcaaagacagattgctgcaatcactaaaccatcttttcgcagcgatggattcg
acagatgcagatgttgtcatctattgcagggacaaggaatgggagaagaagatcactgaagccatatcactaagatcccag
gtagaactactagatgatcacatctcagtggattgcgacattgtacgcgttcatccagacagcagcctggcaggccgaaag
gggtacagcacagtagagggagcactctactcttacctagagggaacaagattccaccaaactgctgtagatatggctgag
atatataccatgtggccaaaacaaactgaagccaacgaacaggtctgcctatatgctctgggggagagtatagagtccgtc
aggcaaaaatgtcccgtagacgacgccgacgcctcattccctccgaaaacagtcccgtgcctatgccgttatgccatgacg
cctgaacgagttgcacgcctacgcatgaatcatactactagcatcatagtgtgctcgtcttttccgctgccgaagtacaaa
atcgagggcgtgcaaaaagtaaaatgttcaaaagcactcttgtttgatcacaacgtaccgtctcgagtgagcccgagaacg
tacaggcctgcggacgaaatcatacagacacctcaaatatcaactgaagcgtgtcaggacgcacaactcgtgcagtcaata
aatgatgaagcagtgccagttccctcggacttagaggcttgtgacgcaactatggactggccctctatcggcaccgtacca
acaagacaaagacacgactcatttgacagcgagtatagttccagaagcaacatacagctagtgacggcggacgtgcatgca
ccaatgtacgcaaattcgctggcgtccagcggaggttcaatgctgtcgctgtccagtgaaccagctcagaacggcataatg
atactacctgactcagaagacacagatagtataagcagagtaagcacaccgatcgccccgcccaggagacgtttgggaagg
accataaatgttacttgtgacgagcgggaagggaaaatactccctatggccagcgacaggttcttcactgctaagccatac
actgtcgcactgagcgtatcaacagcagacataactgcgtaccccatccaggcaccgctaggattgacacaaccacctacc
ctcgaacagatcactttcggagattttgccgaaggtgaaatagacaacctcctgacaggggcattgacatttggggacttc
gagccaggtgaagtggaagagctgacggatagcgagtggtcaacatgctcggacacagatgaagagttacgactagacaga
gcaggggggttga

> RRV-QML

atggcaccctcataccgtgtgcgcaggaccgacatttccgggcacgctgaagaggcggttgtcaatgccgccaatgcgaag
ggcacagtcggtgatggggtttgcagagcggtggcaaaaaaatggccagactccttcaaaggtgccgcgactcccgtgggt
acggccaagttggtacaggccaacggcatgaatgtcatccacgcggtaggcccgaatttctccacggtgaccgaggcagag
ggagacagagagttggccgccgcataccgtgccgtggcgggtatcatcaatgccagtaacattaagagtgtagccatccct
ctgttgtcgacgggagtgttctccggaggtaaagatagagtcatgcagtcactaaatcatctgtttaccgcaatggacacc
acggacgctgacgtagttatctactgtcgcgacaaagcctgggagaagaaaatccaggaggctatcgaccgccgcaccgcc
gtggaattggtatctgaagacatctcactcgagtctgacttgatacgggtgcacccggatagttgcttggtaggcagaaaa
ggttacagcataacagatgggaagctgcattcatacctggaaggtacccgcttccatcagactgcggtggacatggctgag
atatctaccttgtggccgaaacttcaggacgcaaacgagcaaatatgcttgtatgcattgggtgagagtatggacagtatc
agaacgaaatgccctgtcgaggacgccgattcgtccacgcctccaaaaacagttccgtgtctgtgtaggtatgctatgact
gctgagagagtggcgaggctccggatgaacaacactaaggccataattgtgtgctcctccttccctttaccgaagtacagg
attgaaggcgtccagaaggtcaagtgcgaccgagtgctgatttttgaccagacggtgccatctctggttagcccaaggaaa
tacataccagccgccgcctccatgcacgcagataccgtgagtttggactctacagtattgcacgcagataccgtgagtttg
gattctacggtatctacaggatctgcgtggtcattcccatctgaggccacgtatgagaccatggaagtagtagcagaggtg
caccactcggaaccaccagtcccaccaccgcgcagacgtcgtgcgcaggtgacgatgcaccaccaggagctgttggaagtc
tccgacatgcacaccccgattgcggcaagggtcgagatccctgcgtacgataccgctgttgtagtggagagagtggcaatt
ccttgcataagcgagtatgcaaccccaataccagcaccgcacgcagcaagggtcgttcccgtgccggcaccgcgcattcag
cgagcgtcgacgtacagagtctctcctacacccacgcctcgcgttctgaaagcctcagtatgcagtgtgaccactagcgct
ggggtagagttcccttgggcacctgaagatttggaggtacttaccgagcctgtgcactgcgaaatgcgcgagccggttgag

ttaccgtgggagccggaggacattgacatccaattcggagattttgaaacacccgacaaaatccaattcggcgacatcgat
tttgaccaattctga

> RRV-T48

atggcaccctcataccgtgtgcgtaggaccgacatttccgggcacgctgaagaggcggttgttaatgccgccaacgcgaag
ggcacagtcggcgatggggtttgcagagcggtggcgagaaaatggccagactccttcaaaggtgccgcgactcccgtgggt
acggctaagttggtacaggccaacggtatgaatgtcatccacgcggtaggcccgaatttctccacggtgaccgaggcagag
ggcgacagagagttggccgccgcataccgtgccgtggcgggtattatcaatgctagtaacattaagagtgtagccatccct
ctgttgtcgacgggagtgttctccggaggtaaagatagagtcatgcagtcactaaatcacctgtttaccgcaatggacacc
acggacgctgacgtagtcatctattgccgcgacaaagcctgggagaagaaaatccaggaggctatcgatcgccgcaccgcc
atggaattggtatctgaagacatctcactcgagtctgacttgatacgggtacacccagatagttgcttggtaggcagaaaa
ggttacagcataacagatgggaagctgcattcatacctggaaggtacccgctttcatcagactgcggtggacatggctgag
atatctaccttgtggccgaaacttcaggacgcaaacgaacaaatatgcttgtatgcattgggtgagagtatggacagcatc
agaacgaaatgccctgttgaggacgccgattcgtccacgcctccgaaaacagttccgtgtctgtgtaggtacgctatgact
gctgagagagtggcaagacttcggatgaacaacactaaggccataattgtgtgctcctccttccctttaccgaagtacagg
attgaaggcgtccagaaggtcaagtgcgaccgagtgctgatttttgaccagacggtgccatctctggttagtccaaggaag
tacataccagccgccgcctctacgcacgcagataccgtgagcttggattctacagtatccacaggatccgcgtggtcattc
ccatctgaggccacgtatgagaccatggaagtagtagcagaggtgcaccactcggaaccaccagtcccgccaccgcgcagg
cgtcgtgcgcaggtgacgatgcaccaccaggagctgttggaagtctctgacatgcacaccccgattgcggcaagggtcgag
atccccgtgtacgataccgctgttgtagtggagagagtggcaattccttgcacaagcgagtatgcaaaacccataccagca
ccacgggcagaaagggtcgtacccgtgccggcaccacgcattcagcgagcgtcgacgtacagagtctctcctacacccacg
cctcgcgttctgagagcctcggtatgcagtgtgaccactagcgctggggtagagttcccttgggcgcctgaagatctggag
gtactcaccgagcctgtgcactgcaaaatgcgcgagccggttgagttaccgtgggagcctgaggacgttgatatccagttc
ggagattttgaaacatccgacaaaatccaattcggcgacatcgattttgaccaattctga

> TAFV

atggccccatcctacacagtggtcaggggcaacattacggccacccgcgcacatgccgtcgtcattccagtcacaccagac
cacaaagacggcgtgtacagatcctgttctaaaaaatggggaccgcttccacctatggagtggtcggaaggaggtacgctg
ttctcgcccggcacgcctgccacactgcaggtatgcgtacccgccctccataacgccgactcgaccgcaactcagcaggcg
taccgtgccatagcaaaggtggtcgttgacgaacaaataccgtccctttcgctcccggtattagcaatcaagaaaaatggc
gcatctgatgtcgtgacggagtcactcaaccatctcgtaaccgcattggaccaaactgacgcggacgtcaccatatactgc
ctggataagggacggcaccgaaagatcgcggaagttatcgcacgcaaagaggcagtaacggagctagtggacgacgacctc
gagatcgacgaagagctgacttgggtacacccggacagttgccttaagaaccgtaacggcttcagtaccagcaaaggcaaa
ctgtactcctacctcgagggtacgaaattccaccaaatggcgaaggactttgctgaaataaggtcgctgttcccatcggaa
gcagaggccaacgaacacatttgctcactaatactaggggagactattgaaggtatccgcgagagctgcccggtatccgac
aaccctccgtctacccccccaagacagtaccgtgcttgtgcatgtacgccatgacaccggagagagccttgcgcctcaaa
agcaattccgtgacgcagatcacagtttgctcctcattcaccttagggaggcatcacatcaaaggggtccagaagatagca
tgcttgtcaccgatgctcttcaaccccgcaccgctcgtcgcccgcaccatccggaagcctccagcgctggccacacgggca
cctatcgatctgcagatgccagcgacacggcccacgcctgcacggttggtgcccacgcgcagagccccgtcacctcccagc
tacaaaaccgttaagcgacccacagcaccgcctccgccgccgcctacctcgcgcccgccgaccccgtcccggcaccgagg
ctgtcgctccttcagacgcctgtcccggcgccgcgccgccacgttagcccccgccttcggaggaaggcagcctgcaacta
cctgctacctccgaggacaccgtcagcttctcattcggggacttcagcgaagatgaggtttcatccctgacgggaggagag
tattga

> UNAV

atggcaccagcctatcgggtgatcagggctgatattgcgaccagcaaggagcaggctgtggtgaatgcggcaaaccatcgt
ggcatcatcggcgatggagtatgtcgtgcgatcggtcgcaaatggccacaggcgttcgtgaattctgctacaccagtgggt
actgcaaagacagtggagtgtgacggtgttcatattatccatgccgtcggacctaatttcagtaacacatcagagcaggaa

ggtgacagagacctggcggccgcctacagagctatcgcagccgaagtcaaccggcttcgtataaccacggtggcaatcccg
ctcctttcgacaggggcgttcagcgccggcaaagacagagtccgccaatcactgaaccacctcttcacagcactagataca
acagatgcagatgtaacgatctactgcagggacagagactgggaaaagaggattaaggacgccatcgacatgaggaccgcg
caagagctggtcagtgacaccacggacctggaagtggacctggttagagtccaccccgacagcagcctagccggccggaga
ggctacagcaccactgatgggtcactatactcctacttggaaggcacaaaatttcaccaggtgtcaatagatatggcagaa
ataataaccatgtggcctaaattggcagaagcaaacgagcagatatgtctatacgctatgggcgagacaatggacaatatt
agagctagatgccctgtggacgattgcgaatcgtccacaccaccaaaaacagtgccctgtttgtgcaggtacgccatgacc
gcggagcgcgtgacacggctaaggatgcaccatagcaagggattcacagtctgctcatccttccaactgccgaagtaccgc
atagagggcatacagagagtcaagtgtgacaaagttttgctgtttgacccgacggtaccttccttagtgagtcctagaaga
tacgtggagcgccgaacctcactgatatctgcgggcagcgacgaacttgtcccgcactcacccagtcgcgagtcagtgaat
gcagagaccatgtcgacggatggggagttcgtcccgcggtcgctcagccatgcacctgtggacttgggttctaacagccat
ttgtatgtatctatgcacaatgttaccgcaatgtcccctcccattccaccacccaggccgaagagagcacgacgccttgct
gctctgttggcgcgtcccatcccggcgcccaggactcgaataacggtcacagcagacatacatgtaccagcagaaccacct
gtgcccatccccagaacacgcgtccgcagaccatcggaggactcggcagtttctttaccgtgggacctcaaggaaacgccc
ctaccggcaggggcgccggtgcctgccccaagaagtgttcgcagaccatcagagggcactgtagtcactggttcaatggtt
tccttgccgtgggatccagaggatattgatttggaatttgaggcgcctttggatgatatcatcaccttcggagactttacc
gaagaagaggtggcgtctctcgacagtgaaggagtggaaatgccatgggatgcacagcccgagattaggcatatgcctctg
actttcggagatttctctgatgacgagtgggaaaccgtaagctcttcatcatcctga

> VEEV

atggcaccctcatatcatgtggtgcgagggatattgccacggccaccgaaggagtgattataaatgctgctaacagcaaa
ggacaacctggcggaggggtgtgcggagcgctgtataagaaattcccggaaagcttcgatttacagccgatcgaagtagga
aaagcgcgactggtcaaaggtgcagctaaacatatcattcatgccgtaggaccaaacttcaacaaagtttcggaggttgaa
ggtgacaaacagttggcagaggcttatgagtccatcgctaagattgtcaacgataacaattacaagtcagtagcgattcca
ctgttgtccaccggcatcttttccgggaacaaagatcgactaacccaatcattgaaccatttgctgacagctttagacacc
actgatgcagatgtagccatatactgcagggacaagaaatgggaaatgactctcaaggaagcagtggctaggagagaagca
gtggaggagatatgcatatccgacgactcttcagtgacagaacctgatgcagagctggtgagggtgcatccgaagagttct
ttggctggaaggaagggctacagcacaagcgatggcaaaactttctcatatttggaagggaccaagtttcaccaggcggcc
aaggatatagcagaaattaatgccatgtggcccgttgcaacggaggccaatgagcaggtatgcatgtatatcctcggagaa
agcatgagcagtattaggtcgaaatgccccgtcgaagagtcggaagcctccacaccacctaccacgctgccttgcttgtgc
atccatgccatgactccagaaagagtacagcgcctaaaagcctcacgtccagaacaaattactgtgtgctcatcctttcca
ttgccgaagtatagaatcactggtgtgcagaagatccaatgctcccagcctatattgttctcaccgaaagtgcctgcgtat
attcatccaaggaagtatctcgtggaaacaccaccggtagacgagactccggagccatcggcagagaaccaatccacagag
gggacacctgaacaaccaccacttataaccgaggatgagaccaggactagaacgcctgagccgatcatcatcgaagaggaa
gaagaggatagcataagtttgctgtcagatggcccgacccaccaggtgctgcaagtcgaggcagacattcacggccgccc
tctgtatctagctcatcctggtccattcctcatgcatccgactttgatgtggacagtttatccatacttgacaccctggag
ggagctagcgtgaccagcggggcaacgtcagccgagactaactcttacttcgcaaagagtatggagtttctggcgcgaccg
gtgcctgcgcctcgaacagtattcaggaaccctccacatcccgctccgcgcacaagaacaccgtcacttgcacccagcagg
gcctgctcgagaaccagcctagtttccaccccgccaggcgtgaatagggtgatcactagagaggagctcgaggcgcttacc
ccgtcacgcactcctagcaggtcggtctcgagaaccagcctggtctccaacccgccaggcgtaaatagggtgattacaaga
gaggagtttgaggcgttcgtacacaacaacaatgacatcttctag

> WEEV

atggctccagcgtacagagtgatcagaggtgacattagcaagagcgctgaccaagctatcgttaatgctgctaatagcaaa
ggtcaaccaggttccggagtgtgcggtgcactgtaccgaaaatggccggctgcttttgatagacagccaatagctgtcggg
acggctagacttgtgaagcacgaaccgctcatcatacatgctgtaggacccaatttttctaagatgccggaaccggagggc
gaccttaagctcgcagctgcctacatgagcatagcgtccatcgtcaacgctgagcggattactaaaatatcagtaccgcta
ctgtcaactggcatctattctggtggcaaagatcgagtgatgcaatcattgcatcacctgttcactgctttcgacactacg
gatgccgatgtcaccatatattgcttggataaacaatgggagaccaggataatcgaggccattcaccgcaaagaaagcgtc
gaaatactggatgatgatgacaagccagtagacatcgacttggtcagggtccacccaaacagctctttggcaggcagacca
ggttactccgtcaatgagggcaagctgtattcatacctggaaggtacacgattccatcagaccgccaaggacattgccgaa
atccatgcaatgtggcccaacaaatctgaggctaatgagcagatttgcttgtacatcctgggggagagtatgtccagcatc

cgctccaaatgcccagtagaggagtcagaggcgtctgctccacctcacacacttccatgcctgtgtaattacgctatgacg
gctgagcgcgtatacaggttgcgctctgcgaagaaagaacagttcgccgtatgctcatcattcccgttgccgaagtacagg
atcacaggcgtgcagaagctacaatgcagcaaaccagtcctgttctcaggcgtcgtaccaccggctgtacaccccaggaag
tacgcggaaataattctagaaacgccaccatcgccaacaacgacaaccgtaatatgtgaaccaactgtgccagaacgtata
cccagtccggtgatttctagagcaccaagtgcggaatcactgctatcgtttggcggcgtctcgttctctagctctgccaca
cgctcgtcaaccgcctggagcgactatgacaggcggtttgtggttacagctgacgtgcatcaagcgaacacgtctacgtgg
agcatccctagtgctcctggcttggacgtccagctgccttctgacgatactgattcccactggagtattccaagtgcatca
ggctttgaagtgagaacaccatctgtacaggacctaactgcagagtgtgcgaggcctcgtggactggccgaaataatgcaa
gacttcaatactgctcctttccagtttctttcggaccacagaccagtaccagcaccacggagacgccccatcccatcacct
agatcgacggcttccgcacctccagttccaaagccacgcagaactaagtaccaacaaccaccaggagtcgctagggcgatc
tcagaagcggagttggacgagtacatacgtcaacattccaactgacatatg

> WHAV

atggcgccatcgtacaaatcaaggagaggaaacatcatcgaatgcaccgaagaagccgtcgtgaacgctgccaacgcacta
ggacgccccggagaaggggtctgcaaggcgatttacaagaagtggccgaacagcttcaccggttccgcaacagaagtaggg
actgcaaaaatgaccacaagcctaggcaagaaagtcatacatgccgtcggaccggattttaagaagcactctgaagaagaa
gcccttaaactgctgcagaatgcctaccacgccatcgcagatattattaatgagaacaacatcaaatcagtggccattcca
ttgctatcaactggtatatacgctgcagggaaggacagactagagacttctttgcactgtttgaccacagcgatggacagg
acggacgccgacgtaacggtatactgccttgacaagaaatggcagcagcgaattgacgcagtccttagattgaaagaagag
gtaacggagctaaaagacgacgacatggaaattgatgaggagctggtttggatccaccctgacagctgtttgaaaggacgt
aaaggctttagcaccaccaaaggcaaactgtattcatacttcgaaggaactaaatttcaccaggcagcgaaagacatggca
gaaatcaatgtattgtttccagacaccattgaggctaacgagcaaatctgtatgtatatccttggagaaagcatggaagct
atccgcgaaaaatgccccgtcgactacaacccttcgtcaagtccgccaaaaaccttaccctgcctgtgcatgtatgctatg
acacctgagagggtgcatagactcagaagcaacaatgtcaaagaaattacggtatgctcctcgactccacttccaaaacat
aaaatcaagaacgtacaacgaatccagtgttcaaaaatcgtcttgtttaatccccagactccagcttttgtacctgcacgt
aagttcatagaaaccgaacccaaagaaacagaagacgatgcggctcagccggacccgacaccggcagtgcaggcgagtgtt
tcgaccccggtcccacaacgtcagcaagacccgttagagttgataatatccgcagactctttaaccgaagtaaacgacacc
tctgacgacatttccgacatacccttgacacatctgtatatgctagtacttcctcactgagctcggttttggactgccac
aatgtagtcgaggtcgaggcggaaattcacgtcgtcccgcagactccggtggcaccgccgagaaagaagaagttagcacgt
ttagcggcgctatcaagagcatctagcatttcctccatcgaatccaacccaccaatcacttttggatcatttgaggatgga
gaaatagacaacttgcagaagaagtgcacttcagaaccatttatgttcggctcgttcgaaccaggcgaagtcaacagcctg
atagaaaccaggtcggagccaccacgtagggggcgcagacgtcgcaacaagaaccgacaggagtattga
